# Efficacy and Safety of Bioresorbable Vascular Scaffolds Versus Contemporary Drug-Eluting Stents in Coronary Artery Disease: A Systematic Review, Meta-Analysis, and Trial Sequential Analysis

**DOI:** 10.31083/RCM51636

**Published:** 2026-10-20

**Authors:** Yue Yu, Ke Shang, Yuemiao Jiao, Sanshuai Chang, Yang Li, Guangyuan Song, Chengqian Yin

**Affiliations:** ^1^Interventional Center of Valve Heart Disease, Beijing Anzhen Hospital, Capital Medical University, 100029 Beijing, China

**Keywords:** bioresorbable vascular scaffolds, drug-eluting stents, coronary artery disease, percutaneous coronary intervention, meta-analysis, trial sequential analysis

## Abstract

**Background:**

Bioresorbable vascular scaffolds (BVS) were developed to address some limitations associated with permanent metallic drug-eluting stents (DES), but the comparative safety and efficacy of these scaffolds remain uncertain.

**Methods:**

We conducted a systematic review and meta-analysis of randomized and observational studies comparing BVS with contemporary DES in patients with coronary artery disease. The primary outcome was the composite clinical endpoint at 1-year. Secondary outcomes included mortality, myocardial infarction (MI), revascularization, thrombosis, and angiographic measures. Outcomes at 2, 3, and 5 years were analyzed separately. Trial sequential analysis (TSA) was used to assess the robustness of the evidence for the primary outcome.

**Results:**

A total of 51 studies (69 reports) were included. At 1 year, BVS was associated with a higher risk of the composite clinical endpoint than DES (risk ratio (RR) 1.11, 95% confidence interval (CI 1.00–1.22)), mainly driven by increased risks of MI, target lesion revascularization, and thrombosis. In contrast, all-cause and cardiac mortality did not differ significantly between groups. TSA showed that although the cumulative Z-curve reached the required information size, the Z-score did not cross the trial sequential monitoring boundary, indicating that the evidence for the primary endpoint remained inconclusive. During mid- and long-term follow-up, BVS remained associated with higher risks of ischemic events and repeat revascularization, without a survival benefit. Angiographic analyses showed greater late lumen loss, smaller minimal lumen diameter, and greater diameter stenosis with BVS.

**Conclusions:**

Compared with contemporary DES, BVS appears to confer a higher ischemic risk without a long-term mortality benefit. Contemporary DES remains the preferred revascularization strategy.

## 1. Introduction

Coronary artery disease (CAD) continues to be one of the principal causes of illness and death across the globe [1]. Although substantial progress has been made in preventive strategies and pharmacological treatment, the total number of people living with CAD has still risen over the past several decades, largely as a result of population aging and the increasing burden of cardiometabolic risk factors [2,3,4].

Percutaneous coronary intervention (PCI) is now the dominant revascularization approach for patients with obstructive CAD [5]. The evolution from plain balloon angioplasty and bare metal stents to modern drug-eluting stents (DES) has markedly lowered the incidence of restenosis and the need for subsequent revascularization procedures [6,7]. Nevertheless, the permanent implantation of metallic stents remains associated with several unresolved limitations [8]. Chronic exposure of the vessel wall to a permanent metallic scaffold may sustain local inflammatory responses, promote delayed neointimal proliferation and neoatherosclerosis, and increase the risk of late and very late stent thrombosis [9]. In addition, permanent stent caging may impair physiological vasomotor function and adaptive vascular remodeling, while metal hypersensitivity has also been proposed as a potential contributor to adverse vascular responses [10,11]. From a clinical standpoint, the need for prolonged antiplatelet therapy after metallic stent implantation may increase long-term bleeding risk, particularly in patients with high hemorrhagic vulnerability [7].

These limitations have stimulated the development of revascularization strategies based on the concept of leaving no permanent implant within the coronary artery. Drug-coated balloons (DCB) and bioresorbable vascular scaffolds (BVS) are the two main therapeutic forms of this strategy. DCB have been incorporated into clinical practice guidelines and expert consensus statements for selected indications, including small-vessel disease, bifurcation lesions, and in-stent restenosis [5,7,12,13]. However, their role in large vessels and in complex or heavily calcified lesions remains less well established. In addition, the absence of a permanent scaffold may predispose the vessel to early recoil or acute closure in certain anatomical settings. Although earlier meta-analyses suggested that long-term outcomes with DCB may be comparable to those achieved with second-generation DES [14], recent large randomized trials conducted in unselected target vessels failed to demonstrate noninferiority, thereby reinforcing DES as the default strategy for unselected coronary lesions [15].

BVS were developed to address some of these limitations by providing temporary mechanical support and local drug delivery during the early healing phase, followed by complete scaffold resorption [16]. In theory, this strategy could restore vascular physiology, reduce chronic inflammatory stimuli, and eliminate the long-term consequences of permanent intracoronary metal [9,16]. Early single-arm studies of first-generation poly-L-lactic acid (PLLA)-based scaffolds reported favorable short-term clinical outcomes, leading to rapid clinical adoption and the initiation of large randomized trials [17]. However, subsequent randomized studies and large registries demonstrated higher rates of scaffold thrombosis and MI with BVS than with contemporary DES, raising major safety concerns and ultimately leading to the withdrawal of the first-generation Absorb scaffold from routine clinical use [18,19].

In parallel, newer-generation bioresorbable platforms, including magnesium-based and iron-based scaffolds, have been developed to improve mechanical performance and vascular healing [20,21,22]. At the same time, evidence comparing BVS with contemporary DES has accumulated from randomized trials and observational studies, with heterogeneous findings across follow-up durations, scaffold materials, lesion characteristics, and clinical presentations [23]. Although early adverse ischemic events have been consistently reported with first-generation polymer-based scaffolds, uncertainty persists regarding longer-term clinical outcomes, angiographic performance, and whether newer scaffold platforms may modify the overall risk profile [18,24,25].

Despite evidence from randomized trials and observational studies, the comparative efficacy and safety of BVS versus contemporary DES remain incompletely defined because of heterogeneity in follow-up duration, scaffold platforms, lesion characteristics, clinical presentation, and endpoint definitions. Although an increased early ischemic risk has been reported with first-generation polymer-based scaffolds, the consistency and longer-term implications of these findings, as well as their applicability to newer scaffold platforms, remain uncertain. On this basis, we performed a systematic review and meta-analysis to evaluate the comparative efficacy and safety of BVS and contemporary DES in patients with CAD. The primary endpoint was the composite clinical outcome at 1 year, whereas secondary analyses focused on longer-term clinical events and angiographic findings. In addition, trial sequential analysis was undertaken to further determine the strength and reliability of the evidence for the primary endpoint.

## 2. Methods

### 2.1 Search Strategy

This systematic review and meta-analysis was performed in accordance with PRISMA recommendations. A thorough search of PubMed, Embase, Web of Science, and the Cochrane Library was carried out from the inception of each database through December 16, 2025. The search strategy incorporated terms associated with bioresorbable scaffolds, drug-eluting stents, and coronary artery disease. Full search details for each database are presented in **Supplementary Table 1**. In addition, the reference lists of all eligible articles were manually reviewed to identify any further relevant studies.

### 2.2 Study Selection Criteria

Studies were considered eligible if they met the following criteria: (1) included adult patients with coronary artery disease; (2) directly compared bioresorbable scaffolds with drug-eluting stents; (3) reported at least one clinical efficacy or safety outcome; (4) were designed as randomized controlled trials (RCTs) or observational cohort studies; and (5) provided extractable outcome data at one or more predefined follow-up time points.

Studies were excluded if any of the following conditions applied: (1) a noncomparative design or lack of a drug-eluting stent control group; (2) publication types such as reviews, meta-analyses, conference abstracts, case reports, editorials, or correspondence; (3) inadequate information for outcome extraction; (4) duplicate reports or studies based on overlapping cohorts that did not provide additional outcome data; or (5) animal experiments or *in vitro* studies.

For studies reporting outcomes at multiple follow-up durations, clinical endpoints were extracted separately for each reported time point. Short-term and long-term outcomes were analyzed independently to avoid duplication of patient data across analyses.

### 2.3 Study Outcomes

The primary outcome of interest was the composite clinical endpoint assessed at 1 year. If a study reported more than one composite endpoint, priority was given to the device-oriented composite endpoint for data extraction. When this endpoint was not available, major adverse cardiovascular events or the patient-oriented composite endpoint was selected instead.

For studies reporting multiple composite endpoints or multiple follow-up durations, outcome data were extracted according to a predefined hierarchy. Composite outcomes at 1 year were prioritized, followed by those reported at 2, 3, and 5 years. When more than one composite endpoint was available at the same follow-up time point, the device-oriented composite endpoint was selected in preference to other definitions.

Secondary outcomes consisted of the individual elements of the composite endpoints, including all-cause mortality, cardiac mortality, myocardial infarction (MI), target vessel myocardial infarction (TV-MI), target lesion revascularization (TLR), and target vessel revascularization (TVR). These endpoints were extracted separately and analyzed on the basis of the follow-up periods reported in the original studies.

### 2.4 Study Selection and Data Extraction

Two reviewers, Yue Yu and Ke Shang, independently screened all identified records first on the basis of titles and abstracts, and then evaluated the full texts of potentially eligible studies. Any disagreements were settled through discussion in accordance with the prespecified inclusion and exclusion criteria.

Using a predefined standardized form, the same two reviewers independently extracted the relevant data. Differences between the two reviewers were resolved by discussion, and any remaining disagreements were further adjudicated by a third reviewer, Yue Miao Jiao. The extracted data included study design, population characteristics, lesion type, device details, follow-up duration, endpoints, and outcome definitions (Table 1, Ref. [26,27,28,29,30,31,32,33,34,35,36,37,38,39,40,41,42,43,44,45,46,47,48,49,50,51,52,53,54,55,56,57,58,59,60,61,62,63,64,65,66,67,68,69,70,71,72,73,74,75,76]). Baseline demographics and cardiovascular risk factors, clinical history and presentation, target lesion characteristics, and procedural variables are summarized in **Supplementary Tables 2-5**. When more than one publication described results from the same study population, data were extracted at the level of the individual study, and preference was given to the publication providing the longest follow-up.

**Table 1.** **The characteristics of the included studies**.

Study/First author	Year	Design	Target lesion	Number	BVS type	BVS brand	DES type	DES brand	Clinical follow-up	Primary endpoint	MACE definition
Tommaso Gori [29]	2014	cohort	ACS	253	PLLA	Absorb	EES	XIENCE	1 month	MACE	All-cause death, non-fatal MI, or reintervention
BVS-EXAMINATION [30]	2015	cohort	STEMI	580	PLLA	Absorb	EES	XIENCE	1 year	DOCE	Cardiac death, TV-MI, or TLR
Bernardo Cortese [31]	2015	cohort	STEMI	605	PLLA	Absorb	EES		6 months	DOCE/POCE	All-cause death, vessel-related MI, or TLR
Charis Costopoulos [32]	2015	cohort	All-comers	184	PLLA	Absorb	EES	XIENCE	6 months	MACE	All-cause death, follow-up MI, or TVR
ABSORB III [33]	2015	RCT	De novo selected, non-ACS, non-complex coronary lesions	2008	PLLA	Absorb	EES	XIENCE	1 year	TLF	Cardiac death, TV-MI, or ID-TLR
ABSORB China [34]	2015	RCT	De novo selected, non-ACS, non-complex coronary lesions	475	PLLA	Absorb	EES	XIENCE	1 year	LL	Cardiac death, MI, or ID-TLR
Hiroyoshi Kawamoto [35]	2015	cohort	De novo selected, non-ACS, non-complex coronary lesions	499	PLLA	Absorb	SES	Cypher, Cordis Corporation	1 year	MACE	All-cause death, follow-up MI (>48 h post-index), or TVR (30-day and 1-year)
ABSORB Japan [36]	2015	RCT	De novo selected, non-ACS, non-complex coronary lesions	400	PLLA	Absorb	EES	XIENCE	1 year	TLF	Cardiac death, TV-MI, or ID-TLR
ABSORB II [27]	2015	RCT	De novo selected, non-ACS, non-complex coronary lesions	501	PLLA	Absorb	EES	XIENCE	1 year	vasomotion/MLD	Cardiac death, all MI, or ID-TLR
BONITO [37]	2016	cohort	CTO	537	PLLA	Absorb	2nd DES		703 days	TVF	Cardiac death, TV-MI, or ID-TLR
Daniel Dalos [38]	2016	cohort	Mixed population	59	PLLA	Absorb	EES	XIENCE	6 months	SEI/SSI	NA
José de Ribamar Costa Jr [39]	2016	cohort	De novo selected, mixed stable and ACS (UA), non-complex	1624	PLLA	Absorb	EES	XIENCE	1 year	MACE	Cardiac death, MI, TVF, TLR, or ST
Jiang Ming Fam [40]	2016	cohort	STEMI	302	PLLA	Absorb	EES		18 months	MACE	Cardiac death, any re-infarction, CABG, or ID-TLR
Yoichi Imori [41]	2016	cohort	ACS	430	PLLA	Absorb	EES	XIENCE	2 years	MACE	All-cause death, MI, or ID-TLR
UNDERDOGS [42]	2016	cohort	Coronary lesions requiring overlap	324	PLLA	Absorb	EES		1 year	DOCE	Cardiac death, TV-MI, or TLR
V. F. Panoulas [43]	2016	cohort	Overlapping lesions	140	PLLA	Absorb	EES	XIENCE/PROMUS	1 year	MACE	All-cause death, nonprocedural MI, or TVR
TROFI II [44]	2017	RCT	STEMI	191	PLLA	Absorb	EES	XIENCE	6 months	DOCE	Cardiac death, TV-MI, or ID-TLR
Corrado Tamburino [45]	2016	cohort	All-comers real-world registry/observational cohort	1810	PLLA	Absorb	EES	XIENCE	1 year	DOCE	cardiovascular death, TV-MI, or ID-TLR
Jose M. de la Torre Hernandez [46]	2017	RCT	De novo selected, non-ACS, non-complex coronary lesions	200	PLLA	Absorb	BP-EES	SYNERGY	1 year	fluoroscopy time, median dose-area product, contrast agent volume and periprocedural troponin release	NA
Andrea Cuculo [47]	2017	cohort	Complex lesion cohort	110	PLLA	Absorb	EES	XIENCE	417 days	adverse events	All-cause death, cardiovascular death, ST, or TLR
EVERBIO II [48]	2015	RCT	Elective PCI (non-AMI)	238	PLLA	Absorb	EES/BES	Promus Element/Biomatrix Flex	2 years	LLL	Cardiac death, MI, or TLR
Toru Naganuma [49]	2017	cohort	Bifurcation	323	PLLA	Absorb	EES	XIENCE/PROMUS	2 years	TLF	Cardiac death, TV-MI, or ID-TLR
AIDA [26]	2018	RCT	De novo, non-complex coronary lesions	1845	PLLA	Absorb	EES		2 years	TLF	Cardiac death, TV-MI, or TVR
Carlos M. Campos [50]	2018	cohort	Diabetic patients with selected de novo coronary lesions (non-AMI).	432	PLLA	Absorb	EES	XIENCE	780 days	MACE	Cardiac death, all MI, or ID-TLR
Kugler, Christine [51]	2017	cohort	CTO	29	PLLA	Absorb	EES	XIENCE	1 year	DOCE	Cardiac death, MI not related to a non-target vessel, or TLR
Yaling Han [52]	2018	RCT	De novo lesions	560	PLLA	NeoVas	EES		1 year	in-segment LL	All-cause death, all MI, or any revascularization
Frédéric Maes [53]	2018	cohort	All-comers	236	PLLA	Absorb	DES		1 year	MACE	All-cause death, MI, or TVR
BIORESOLVE-ISR [54]	2018	cohort	ISR	184	PLLA	Absorb	EES	XIENCE	1 year	DOCE	Cardiac death, TV-MI, or ID-TLR
ABSORB IV [55]	2018	RCT	De novo selected, mixed stable and ACS lesions (ACS-enriched)	2604	PLLA	Absorb	EES	XIENCE	1 year	TLF	Cardiac death, TV-MI, or ID-TLR
Giuseppe Tarantini [56]	2018	cohort	Small vessels	674	PLLA	Absorb	EES	XIENCE/PROMUS	2 years	DoCE	Cardiac death, TV-MI, or ID-TLR
ISAR-Absorb MI [57]	2019	RCT	MI	262	PLLA	Absorb	EES	XIENCE	1 year	diameter stenosis	Cardiac death, TV-MI, or TLR
COVER-AMI [58]	2019	RCT	AMI	20	PLLA	Absorb	BP-EES	SYNERGY	1 year	neointimal response	Cardiac death, TV-MI, or ID-TLR
Yizhe Wu [59]	2019	RCT	De novo selected, non-ACS, non-complex	395	PLLA	XINSORB	BP-SES	TIVOLI	1 year	LLL	Cardiac death, MI, or ID-TLR
Piotr Desperak [60]	2020	cohort	STEMI	264	PLLA	Absorb	EES		2 years	DOCE	Cardiac death, TV-MI, or ID-TLR
Alexandre Hideo-Kajita [61]	2020	cohort	De novo selected, non-ACS, non-complex coronary lesions	482	Mg	Magmaris	BP-SES	Orsiro	1 year	TLF	Cardiac death, TV-MI, or ID-TLR
Yongcheol Kim [62]	2020	cohort	AMI	300	PLLA	BRS	EES		1 year	DOCE	Cardiac death, TV-MI, or ID-TLR
Jongkwon Seo [63]	2020	RCT	Diffuse long coronary narrowings	341	Mg	Magmaris	EES	XIENCE	1 year	MACE	Cardiac death, MI, ST, or ID-TLR
COMPARE-ABSORB [64]	2020	RCT	ISR	1670	PLLA	Absorb	EES	XIENCE	1 year	TLF	Cardiac death, TV-MI, or ID-TLR
Andrew Kei-Yan Ng [65]	2021	cohort	all-comers	711	PLLA	Absorb	DES		3 years	MACE	All-cause death, non-fatal MI, ST (definite/probable), or ID-TVR
FUTURE-II [66]	2021	RCT	De novo selected, non-ACS, non-complex coronary lesions	433	PLLA	Firesorb	EES	XIENCE	1 year	LLL	Cardiac death, TV-MI, or ID-TLR
Quentin de Hemptinne [67]	2022	cohort	STEMI	90	Mg	Magmaris	EES	XIENCE	1 year	DOCE	Cardiac death or TV-MI
PRAGUE-22 [68]	2022	cohort	ACS	50	Mg	Magmaris	EES	XIENCE	1 year	LLL	All-cause death, ST, or TV-MI
Piotr Rola [69]	2022	cohort	NSTE-ACS	362	Mg	Magmaris	BP-SES	Ultimaster	1 year	cardiac death, myocardial infarction, in-stent thrombosis	Cardiac death, TV-MI, or TLR
Eriksen Erlend [70]	2023	RCT	STEMI	66	PLLA	Absorb	EES	XIENCE	1 year	MFA	NA
Joo Myung Lee [71]	2023	cohort	all-comers	1003	PLLA	Absorb	DES		2 years	TVF	Cardiac death, TV-MI, or ID-TVR
RIBS VI [72]	2024	cohort	ISR	718	PLLA	Absorb	DCB/DES		3 years	MLD	Cardiac death, MI, or revascularization (TLR/TVR)
ISAR-RESORB [73]	2024	RCT	De novo selected, non-ACS, non-complex coronary lesions	117	PLLA	Absorb	BP-EES	SYNERGY	1 year	percentage diameter stenosis	Cardiac death, TV-MI, or TLR
Adrian Włodarczak [74]	2024	cohort	ACS	362	Mg	Magmaris	BP-SES	Ultimaster	2 years	cardiac causes, myocardial infarction, and in-stent thrombosis	TLF, TV-MI, or TLR
Jun Li [75]	2025	cohort	ACS	128	PLLA	NeoVas	BP-EES	SYNERGY	3 years	DOCE	Cardiac death (sudden), ST, TV-MI, or TLR
BIOHEART-II [76]	2025	RCT	De novo selected, non-ACS, non-complex coronary lesions	445	PLLA	Bioheart	EES	XIENCE	3 years	in-segment LL	Cardiac death, TV-MI, or ID-TLR
IRONMAN II [28]	2025	RCT	De novo selected, non-complex coronary lesions	518	Fe	IBS	EES	XIENCE	2 years	LLL	Cardiac death, TV-MI, or ID-TLR

Abbreviation: ACS, acute coronary syndrome; UA, unstable angina; AMI, acute myocardial infarction; BES, biolimus-eluting stent; BP, biodegradable polymer; BP-EES, biodegradable polymer everolimus-eluting stent; BP-SES, biodegradable polymer sirolimus-eluting stent; BRS, bioresorbable scaffold; BVS, bioresorbable vascular scaffold; CABG, coronary artery bypass grafting; CTO, chronic total occlusion; DCB, drug-coated balloon; DES, drug-eluting stent; DOCE, device-oriented composite endpoint; EES, everolimus-eluting stent; Fe, iron-based scaffold; ID-TLR, ischemia-driven target lesion revascularization; ID-TVR, ischemia-driven target vessel revascularization; ISR, in-stent restenosis; LL, late loss; LLL, late lumen loss; MACE, major adverse cardiovascular events; MLD, minimal lumen diameter; Mg, magnesium-based scaffold; MI, myocardial infarction; MFA, major flow abnormality; NSTE-ACS, non–ST-segment elevation acute coronary syndrome; PCI, percutaneous coronary intervention; PLLA, poly-L-lactic acid; POCE, patient-oriented composite endpoint; RCT, randomized controlled trial; SEI, scaffold expansion index; SSI, scaffold symmetry index; ST, stent thrombosis; STEMI, ST-segment elevation myocardial infarction; TLF, target lesion failure; TLR, target lesion revascularization; TVF, target vessel failure; TV-MI, target-vessel myocardial infarction; TVR, target vessel revascularization; IBS, iron bioresorbable scaffold; NA, not applicable.

### 2.5 Risk of Bias and Quality Assessment

The methodological rigor and potential risk of bias of the included studies were evaluated according to their respective study designs. For randomized controlled trials, the Cochrane Risk of Bias 2 (RoB 2) tool was applied to assess bias across five domains, including the randomization process, deviations from the intended interventions, missing outcome data, outcome measurement, and selective reporting [77]. The overall risk-of-bias judgment for each trial was determined according to the Cochrane Handbook [78]. Individual domain-level assessments are presented in **Supplementary Fig. 1**, and an overall summary of risk of bias across all randomized trials is shown in **Supplementary Fig. 2**.

For observational cohort studies, methodological quality was evaluated with the Newcastle–Ottawa Scale (NOS), which assesses studies in three domains: selection of the cohort, comparability between cohorts, and outcome assessment [79,80]. Each study could be awarded up to nine stars. Detailed NOS ratings for all included cohort studies are presented in **Supplementary Table 6**.

### 2.6 Statistical Analysis

All statistical analyses were conducted in R software (version 4.1.1; R Foundation for Statistical Computing, Vienna, Austria) using study-level aggregated data, with the meta package serving as the principal analytical tool. The main endpoint was the composite clinical outcome at 1 year. Secondary clinical endpoints comprised all-cause death, cardiac death, MI, TLR, TVR, TV-MI, target vessel failure (TVF), and definite or probable device thrombosis.

For binary outcomes, pooled risk ratios (RRs) and their 95% confidence intervals (CIs) were estimated using the inverse variance method. Studies reporting no events in either group were not included in the meta-analysis, as such data do not inform the estimation of relative treatment effects. For studies with zero events in only one study arm, a continuity correction of 0.5 was introduced.

Between-study heterogeneity was evaluated with the Cochran Q test and further quantified by the *I*^2^ statistic. A common-effect model was adopted when heterogeneity was judged to be low (*p* > 0.10 and *I*^2^ <50%); otherwise, a random-effects model was employed [81,82]. The choice of model also considered the degree of clinical and methodological variation among the included studies. In random-effects analyses, the restricted maximum likelihood (REML) approach was primarily used to estimate the between-study variance (τ^2^); when convergence could not be achieved, the Paule–Mandel (PM) method was prespecified as an alternative. The Hartung–Knapp adjustment was used to derive confidence intervals for random-effects models.

For studies reporting multiple follow-up durations, outcome data at 1 year were preferentially extracted for the primary analyses. When 1-year data were unavailable, the longest available follow-up was used, with preference given to the time point closest to 1 year.

Secondary angiographic endpoints comprised late lumen loss (LLL), minimal lumen diameter (MLD), and diameter stenosis (DS). For continuous variables, pooled effects were expressed as mean differences (MDs) with 95% CIs, and the same approach to heterogeneity evaluation and model selection applied to dichotomous outcomes was also used in these analyses. In trials with more than one control arm, angiographic outcomes were combined into a single control group in accordance with the Cochrane Handbook, using weighted means and pooled standard deviations to avoid double-counting of participants.

Prespecified subgroup analyses were performed based on study design, including randomized controlled trials and observational cohort studies, lesion complexity, categorized as non-complex versus complex or special lesions, and clinical presentation, classified as acute coronary syndrome (ACS), non-acute coronary syndrome, or mixed populations. For the composite clinical endpoint, an additional subgroup analysis was performed according to endpoint definition, distinguishing device-oriented composite endpoints (DOCE) from patient-oriented composite endpoints (POCE). In exploratory analyses, drug-eluting stents in the control group were further stratified by polymer type into biodegradable-polymer drug-eluting stents (BP-DES) and durable-polymer drug-eluting stents (DP-DES), and scaffold platforms were stratified by material type. Differences between subgroups were formally assessed using tests for subgroup interaction implemented in the meta package, and the corresponding *p* values were reported where applicable.

For the primary outcome, sensitivity analyses were prespecified according to the type of composite endpoint, specifically DOCE versus POCE, and were also performed using a leave-one-out procedure in which studies were removed one at a time to examine the stability of the pooled estimate for the composite clinical endpoint. For outcomes including at least 10 studies, possible publication bias was evaluated by visual assessment of funnel plot symmetry together with Egger’s regression test, and the results of Egger’s test were quantitatively reported when appropriate.

Trial sequential analysis (TSA) was additionally undertaken for the composite clinical endpoint to determine whether the accumulated evidence was sufficiently conclusive and to reduce the risk of random error caused by repeated significance testing, using TSA software (version 0.9.5.10; Copenhagen Trial Unit, Centre for Clinical Intervention Research, Rigshospitalet, Copenhagen, Denmark). The TSA was performed with a two-sided type I error rate (α) of 5%, a power of 80% (β = 20%), and an anticipated relative risk reduction of 15%. The cumulative evidence was considered conclusive if the Z curve crossed both the conventional significance boundary and the trial sequential monitoring boundary [83,84].

All statistical tests were conducted using a two-sided approach, and statistical significance was defined as a *p* value below 0.05. Forest plots were constructed to visually present the pooled effect estimates together with the extent of heterogeneity.

## 3. Results

### 3.1 Study Selection and Characteristics of Included Studies

As shown in Fig. 1, a total of 1583 records were identified from PubMed (n = 266), Embase (n = 583), Web of Science (n = 496), and the Cochrane Library (n = 238). After removal of duplicates (n = 555), 1028 records were screened, and 313 full-text reports were assessed for eligibility. Of these, 244 reports were excluded, primarily because of non-original research (n = 123), non-human studies (n = 3), ineligible study design (n = 43), or irrelevant outcomes or non-extractable data (n = 75). Ultimately, 51 unique studies represented by 69 reports were included. Several trials contributed multiple publications, including extended follow-up reports beyond the initial primary publication. Accordingly, Table 1 summarizes the study characteristics based on the primary report for each trial, whereas longer-term outcomes were extracted from subsequent publications, when available, and incorporated into the corresponding analyses.

**Fig. 1.**
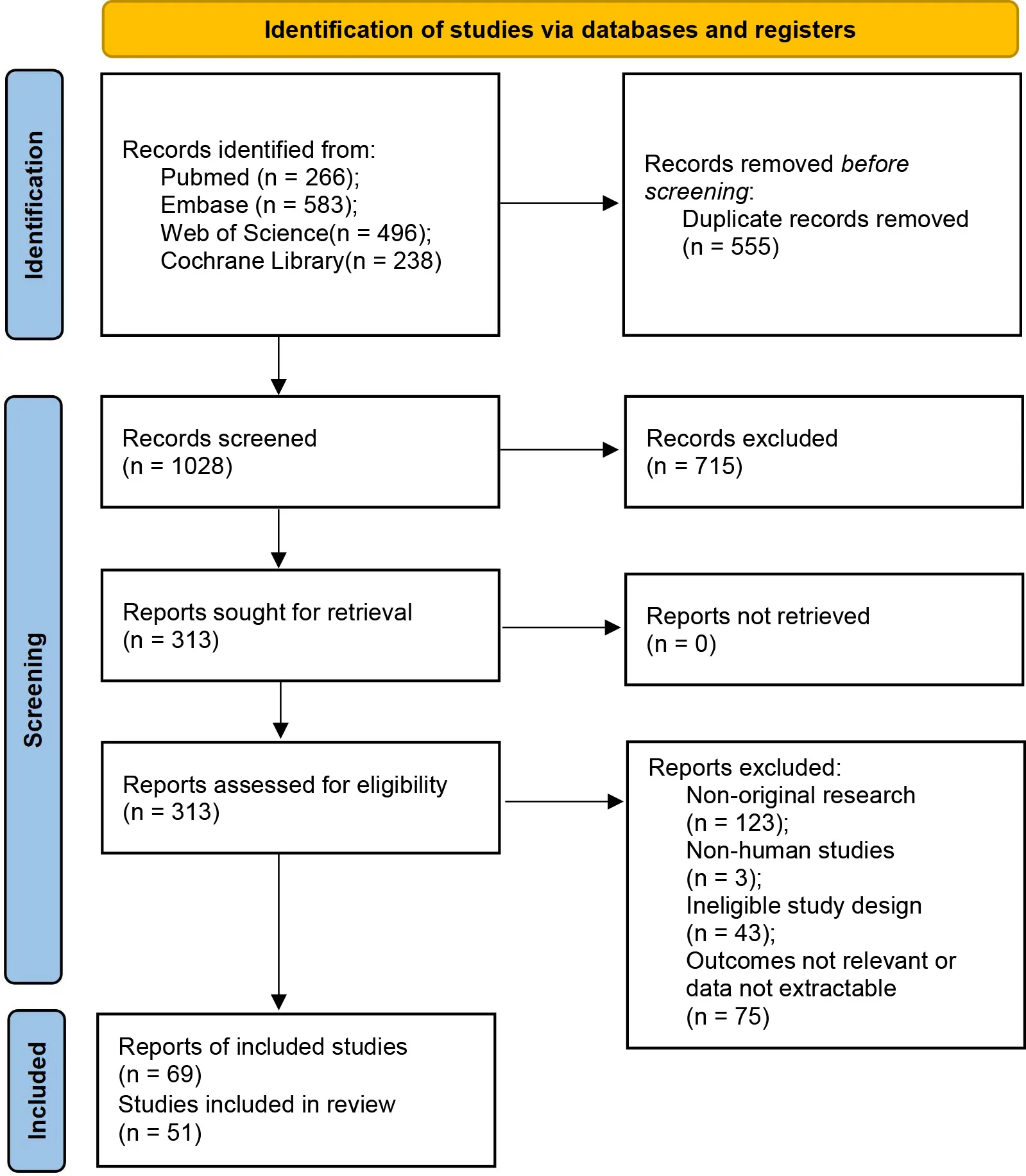
**Study selection process flowchart**.

### 3.2 Characteristics of Bioresorbable Scaffolds and Comparator Devices

Most included studies evaluated PLLA-based bioresorbable scaffolds, predominantly the Absorb platform, with additional representation of newer PLLA-based devices, including NeoVas (Lepu Medical Technology [Beijing] Co., Ltd., Beijing, China), XINSORB (Shandong Huaan Biotechnology Co., Ltd., Jinan, Shandong, China), Firesorb (Shanghai MicroPort Medical [Group] Co., Ltd., Shanghai, China), and Bioheart (Shanghai Bio-heart Biological Technology Co., Ltd., Shanghai, China). Magnesium-based scaffolds were represented mainly by Magmaris, and one recent randomized trial evaluated an iron-based scaffold. Comparator arms primarily used contemporary drug-eluting stents, most commonly everolimus-eluting stents such as XIENCE (Abbott Vascular, Santa Clara, CA, USA) or PROMUS (Boston Scientific Corporation, Marlborough, MA, USA), with biodegradable-polymer DES also included in several studies. One study of in-stent restenosis also included a DCB comparator arm in addition to DES (Table 1).

### 3.3 Endpoints and Study-Level Features

Primary endpoints varied across studies and included clinical composite endpoints (major adverse cardiovascular events (MACE), DOCE or POCE, target lesion failure (TLF), and TVF) as well as angiographic efficacy measures (late lumen loss, minimal lumen diameter, and diameter stenosis). Composite outcomes generally comprised death, MI, and repeat revascularization, although their definitions differed with respect to death (all-cause vs cardiac), MI (overall vs target-vessel-related), and revascularization (TLR vs TVR). Several studies additionally reported thrombosis either as part of a composite endpoint or as a key safety outcome (Table 1). Baseline demographics, cardiovascular risk factors, clinical history and presentation, target lesion characteristics, and procedural parameters are summarized in **Supplementary Tables 2-5**.

### 3.4 One-Year Clinical Outcomes

At the 1 year follow-up, the pooled results indicated that patients treated with bioresorbable scaffolds had a higher rate of the composite clinical endpoint than those treated with drug-eluting stents. Under the common effect model, the pooled risk ratio was 1.11 (95% CI 1.00–1.22) (Fig. 2). Comparable findings were obtained in the random effects analysis, which also yielded an RR of 1.11 (95% CI 1.00–1.22).

**Fig. 2.**
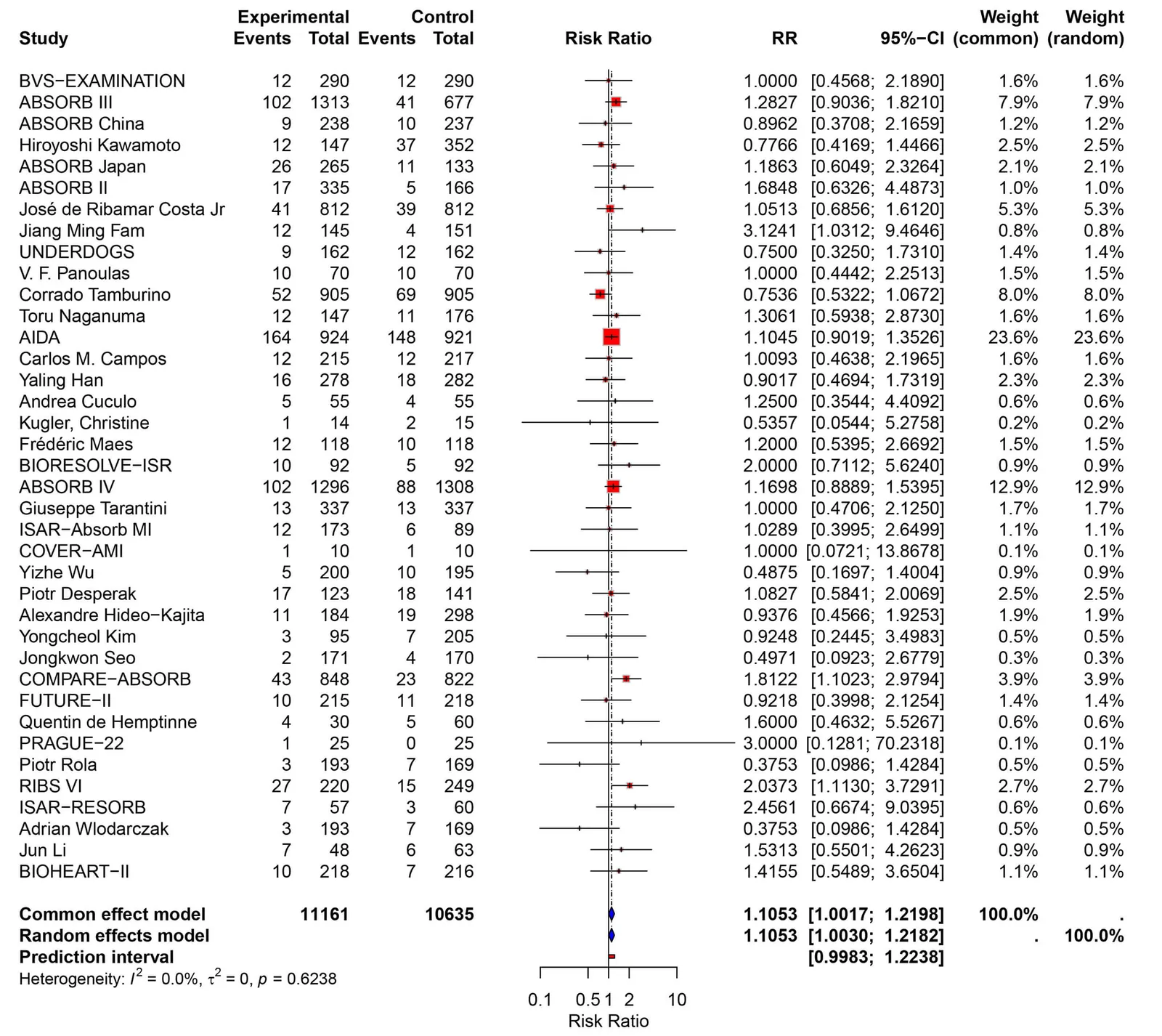
**Forest plot of 1-year composite clinical endpoint comparing bioresorbable scaffolds versus drug**-**eluting stents**.

Heterogeneity across studies was minimal, with *I*^2^ = 0%, τ^2^ = 0, and *p* = 0.624. The prediction interval was 0.998 to 1.224, indicating that although the overall effect was in favor of drug-eluting stents, the strength of this association could differ in future clinical settings.

Trial sequential analysis of the 1-year composite clinical endpoint demonstrated that the cumulative Z curve exceeded the required information size (RIS = 6429), but it did not cross the trial sequential monitoring boundaries for either benefit or harm. The final cumulative Z value was −1.93, which remained below the conventional threshold for two-sided statistical significance. Taken together, these findings indicate that although the information size was sufficient, the currently available evidence is still inadequate to draw a definitive conclusion regarding a difference in the 1-year composite clinical endpoint between bioresorbable scaffolds and drug-eluting stents (Fig. 3).

**Fig. 3.**
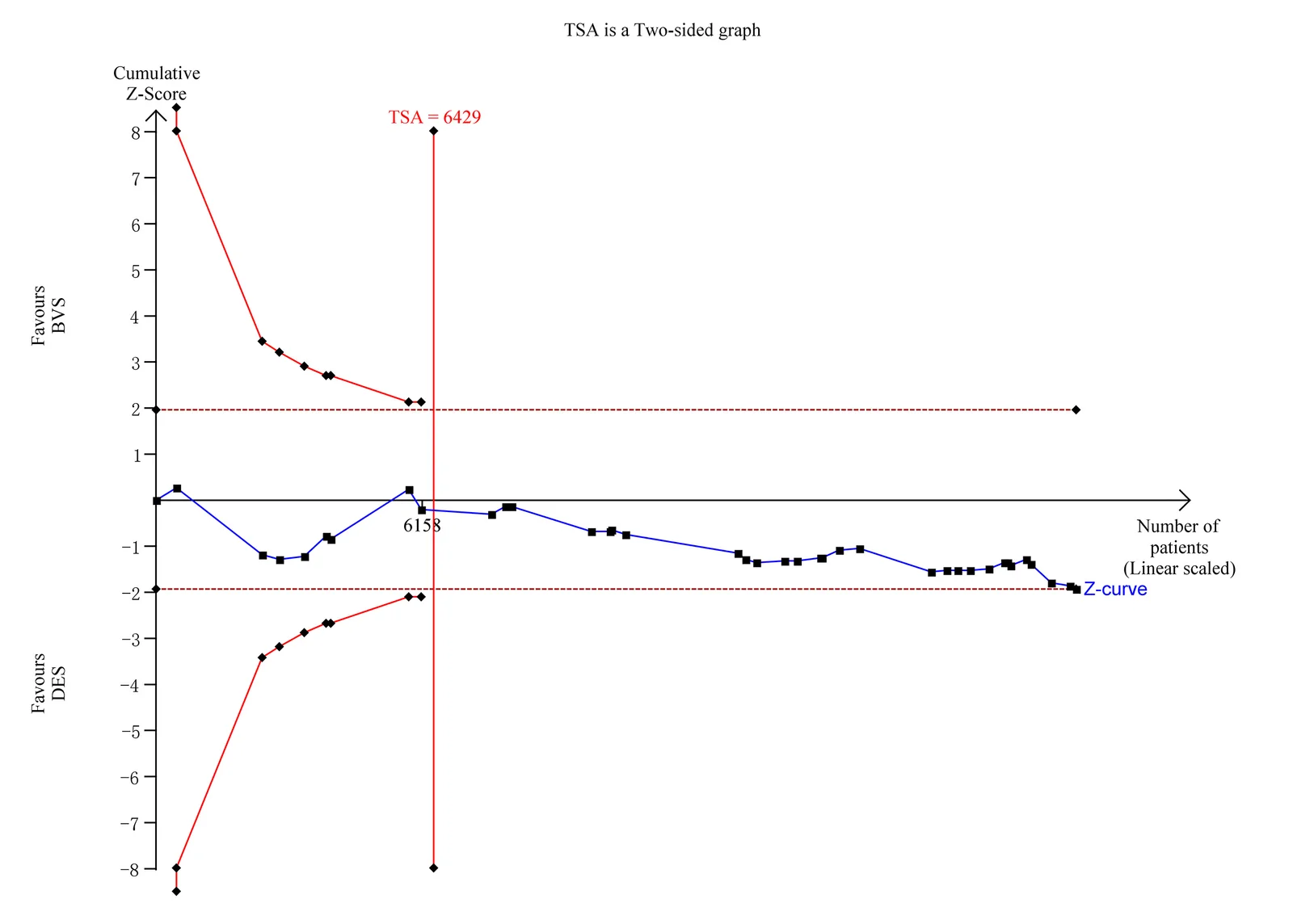
**Trial sequential analysis for 1-year composite clinical endpoint comparing bioresorbable vascular scaffolds versus drug-eluting stents**.

Thrombosis was observed significantly more often in the bioresorbable scaffold group than in the drug-eluting stent group (RR = 1.86, 95% CI 1.33–2.61; Fig. 4). In addition, treatment with bioresorbable scaffolds was associated with increased risks of MI (RR = 1.43, 95% CI 1.22–1.68; Fig. 5) and TLR (RR = 1.21, 95% CI 1.05–1.40; Fig. 6). Because heterogeneity between studies was minimal, the random effects models produced results consistent with those of the primary analyses. Other 1-year outcomes are shown in **Supplementary Fig. 3**. By contrast, no statistically significant differences were identified between bioresorbable scaffolds and drug-eluting stents with respect to all-cause death (RR = 0.97, 95% CI 0.74–1.27) or cardiac death (RR = 0.87, 95% CI 0.65–1.17). Likewise, there were no significant between-group differences in TVR (RR = 1.13, 95% CI 0.97–1.30), TVF (RR = 1.07, 95% CI 0.93–1.23), or TV-MI (RR = 1.20, 95% CI 0.97–1.48).

**Fig. 4.**
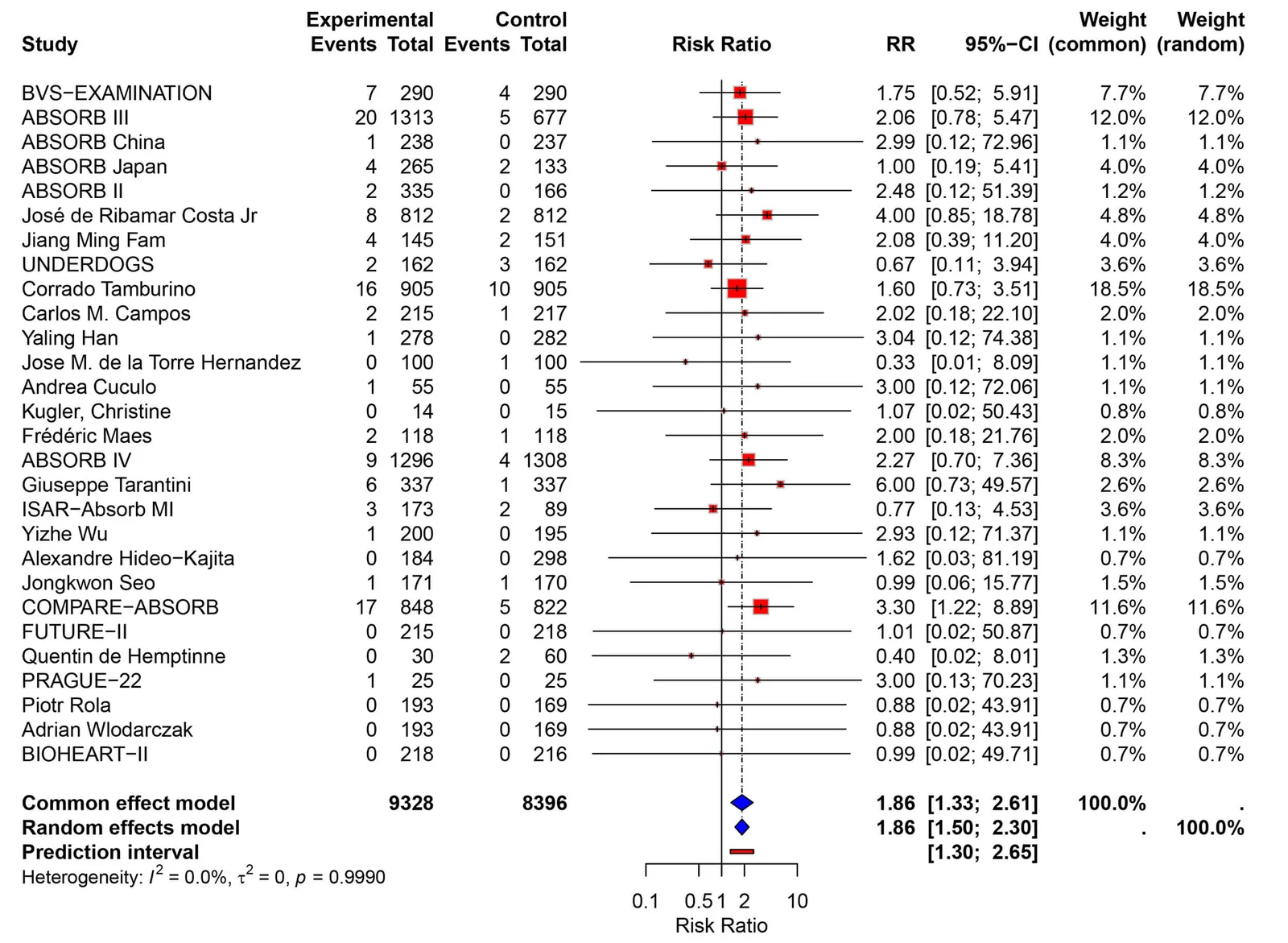
**Forest plot of 1-year scaffold or stent thrombosis comparing bioresorbable scaffolds versus drug**-**eluting stents**.

**Fig. 5.**
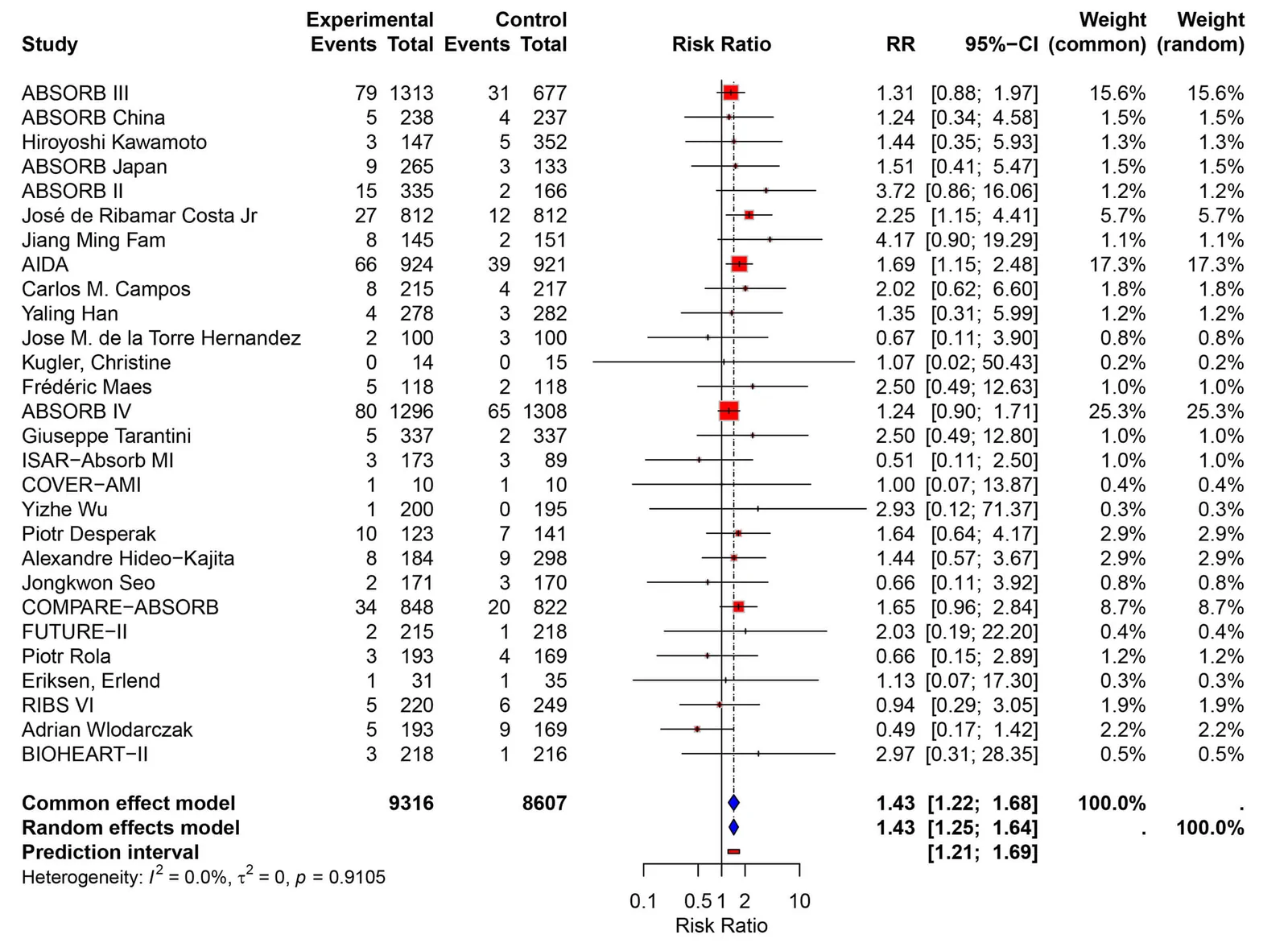
**Forest plot of 1-year myocardial infarction comparing bioresorbable scaffolds versus drug**-**eluting stents**.

**Fig. 6.**
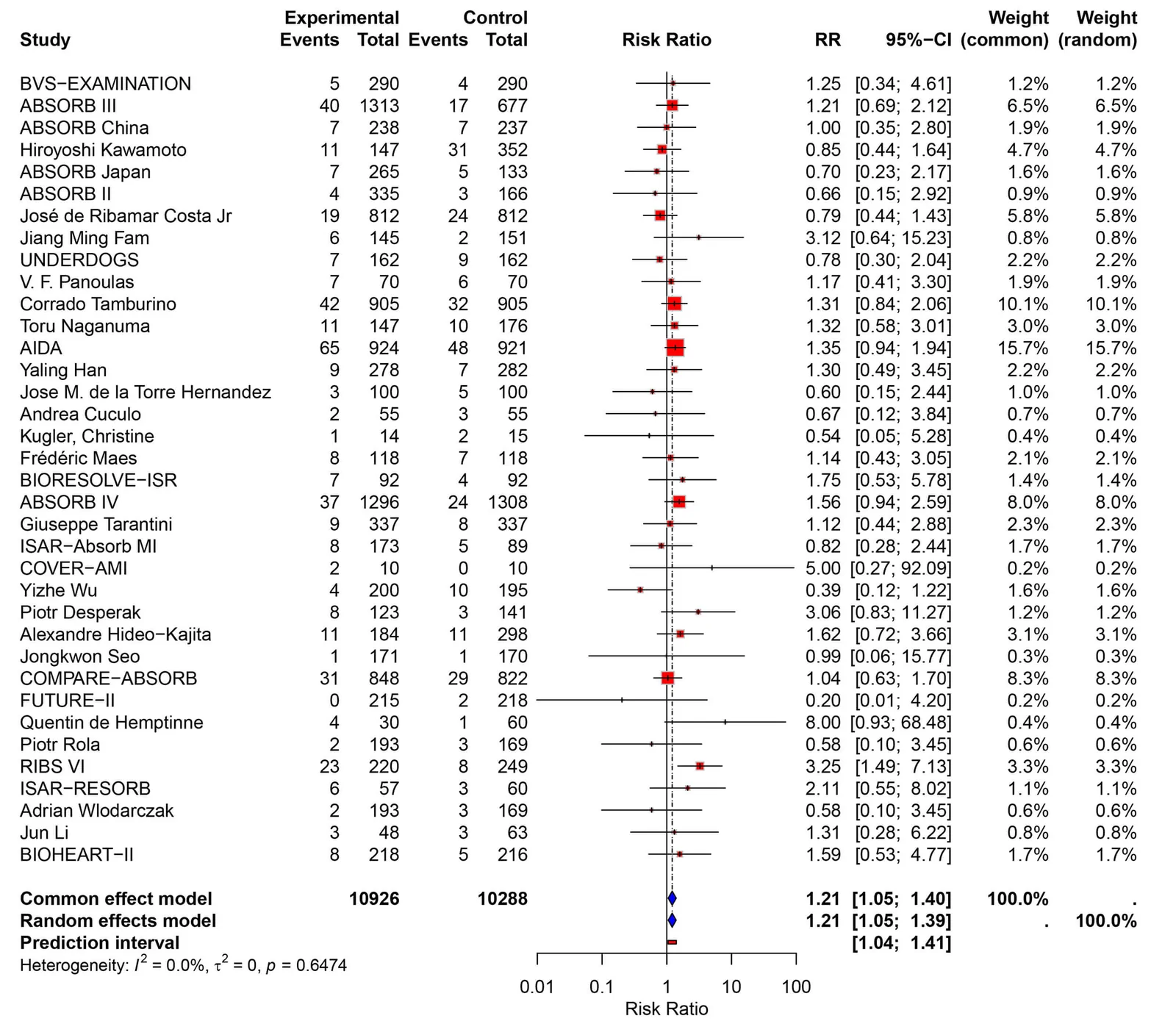
**Forest plot of 1-year target lesion revascularization (TLR) comparing bioresorbable scaffolds versus drug**-**eluting stents**.

### 3.5 Two-Year Clinical Outcomes

At the 2 year follow-up, bioresorbable scaffolds were associated with a greater risk of the composite clinical endpoint compared with drug-eluting stents (RR = 1.28, 95% CI 1.01–1.61; **Supplementary Fig. 4**). Signals related to device safety remained evident, as the risk of thrombosis was still significantly elevated (RR = 2.42, 95% CI 1.52–3.83). In addition, bioresorbable scaffolds were linked to increased risks of TLR (RR = 1.68, 95% CI 1.37–2.06) and MI (RR = 1.37, 95% CI 1.07–1.75), although the pooled estimate for MI was influenced by the statistical model applied.

The pooled results for 2-year mortality and vessel-related outcomes are shown in **Supplementary Fig. 5**. No statistically significant differences between BVS and DES were found for all-cause death (RR = 0.95, 95% CI 0.70–1.28) or cardiac death (RR = 0.82, 95% CI 0.63–1.06) at 2 years. However, BVS was associated with a higher risk of TVR during this period (RR = 1.60, 95% CI 1.17–2.19). No significant between-group difference was observed for TVF (RR = 1.13, 95% CI 0.74–1.71). Although the incidence of TV-MI was numerically greater in the BVS group, the difference did not reach statistical significance (RR = 1.51, 95% CI 0.88–2.58).

### 3.6 Three-Year Clinical Outcomes

Pooled analyses of three-year clinical outcomes are presented in **Supplementary Figs. 6,7**. At 3 years, BVS were associated with a higher risk of the composite clinical endpoint than DES (RR = 1.27, 95% CI 1.10–1.47). The risk of thrombosis was also higher in the BVS group (RR = 2.43, 95% CI 1.05–5.66).

BVS was associated with a significantly higher risk of MI (RR = 1.54, 95% CI 1.21–1.97), whereas no statistically significant difference was found for TLR (RR = 1.27, 95% CI 0.84–1.91). At 3 years, neither all-cause mortality (RR = 1.20, 95% CI 0.89–1.61) nor cardiac mortality (RR = 1.23, 95% CI 0.97–1.56) differed significantly between the two groups. With respect to vessel-related outcomes, BVS was associated with an elevated risk of TVR (RR = 1.30, 95% CI 1.04–1.62), while no significant between-group difference was detected for TVF (RR = 1.08, 95% CI 0.72–1.63). In addition, the incidence of TV-MI was higher in the BVS group (RR = 1.48, 95% CI 1.21–1.82).

### 3.7 Five-Year Clinical Outcomes

At 5 years, BVS remained associated with increased risks of the composite clinical endpoint (RR = 1.20, 95% CI 1.07–1.34), thrombosis (RR = 1.75, 95% CI 1.16–2.64), MI (RR = 1.27, 95% CI 1.10–1.46), and TLR (RR = 1.35, 95% CI 1.16–1.58) (**Supplementary Fig. 8**). By contrast, no significant differences were identified between BVS and DES with respect to all-cause death (RR = 0.98, 95% CI 0.82–1.16) or cardiac death (RR = 0.94, 95% CI 0.73–1.21).

Vessel-related outcomes at 5 years are shown in **Supplementary Fig. 9**. Compared with DES, BVS was associated with higher risks of TVR (RR = 1.22, 95% CI 1.08–1.38), TVF (RR = 1.16, 95% CI 1.04–1.29), and TV-MI (RR = 1.40, 95% CI 1.19–1.65).

### 3.8 Angiographic Outcomes

Pooled analyses of angiographic endpoints are presented in **Supplementary Fig. 10**. Given the substantial between-study heterogeneity across angiographic outcomes (*I*^2^ >50% for all analyses), the results are reported using random-effects models.

Bioresorbable vascular scaffolds were associated with greater LLL than drug-eluting stents, with a pooled mean difference of 0.05 mm (95% CI 0.00–0.11; *I*^2^ = 88.9%). For MLD, bioresorbable scaffolds were associated with a smaller luminal diameter, with a pooled mean difference of −0.18 mm (95% CI −0.26 to −0.09; *I*^2^ = 83.9%). Diameter stenosis was higher in the bioresorbable scaffold group (MD = 4.07 percentage points; 95% CI 1.47–6.66; *I*^2^ = 91.8%).

Prediction intervals for angiographic outcomes were wide and crossed the null, indicating variability in the magnitude of effects across individual studies.

### 3.9 Subgroup Analyses for 1-Year Composite Clinical Endpoint

Prespecified subgroup analyses for the 1-year composite clinical endpoint are shown in **Supplementary Fig. 11**. Overall, the effect estimates were directionally consistent across subgroups, and no convincing evidence of effect modification was identified. After stratification by study design, the pooled RR was 1.23 (95% CI 1.04–1.45) for RCTs and 1.05 (95% CI 0.93–1.18) for cohort studies, without a statistically significant interaction between subgroups (*p* for interaction = 0.137; **Supplementary Fig. 12**). The excess risk of the composite clinical endpoint associated with BVS appeared to be greater in complex or special lesions (RR = 1.37, 95% CI 1.06–1.77) than in non-complex lesions (RR = 1.09, 95% CI 0.95–1.26), although the subgroup interaction did not reach statistical significance (*p* for interaction = 0.129). According to clinical presentation, the estimates were largely comparable among ACS, all-comers or mixed, and non-ACS populations, with no significant subgroup interaction detected (*p* for interaction = 0.895). When analyses were stratified by comparator stent type, the adverse signal was mainly evident in studies using durable-polymer DES (RR = 1.13, 95% CI 1.00–1.27), whereas no clear difference was observed in studies using biodegradable-polymer DES (RR = 0.88, 95% CI 0.59–1.32); however, the interaction test was not significant (*p *for interaction = 0.246). With respect to scaffold type, PLLA-based devices were associated with a higher risk of the composite clinical endpoint (RR = 1.12, 95% CI 1.01–1.24), while magnesium-based scaffolds did not show a statistically significant difference (RR = 0.78, 95% CI 0.48–1.27). Nevertheless, no significant subgroup interaction was found (*p *for interaction = 0.150).

### 3.10 Sensitivity Analyses According to MACE Definition

A sensitivity analysis stratified by composite endpoint definition is presented in **Supplementary Fig. 13**. Among studies using a DOCE, bioresorbable scaffolds showed no clear difference from drug-eluting stents (RR = 1.09, 95% CI 0.97–1.23). Similarly, in studies defining the composite endpoint as a POCE, no statistically significant association was observed (RR = 0.93, 95% CI 0.65–1.32). The test for subgroup differences did not suggest that the effect varied materially by composite endpoint definition (*p* for interaction = 0.389), and the pooled estimate across all studies remained non-significant (RR = 1.07, 95% CI 0.96–1.20).

### 3.11 Publication Bias and Influence Analyses

Visual inspection of funnel plots for the 1-year outcomes showed no marked asymmetry for most endpoints, suggesting no strong evidence of small-study effects (**Supplementary Fig. 14**). Consistently, Egger’s regression test did not detect significant small-study effects for the analyzable clinical outcomes, including the 1-year composite clinical endpoint (*p* = 0.852), death (*p* = 0.802), cardiac death (*p* = 0.367), MI (*p* = 0.957), TLR (*p* = 0.557), TVR (*p* = 0.221), thrombosis (*p* = 0.232), and TV-MI (*p* = 0.542) (**Supplementary Table 7**). For TVF, the Egger test result should be interpreted cautiously because fewer than 10 studies were available. Leave-one-out analyses indicated that the pooled estimates were not materially driven by any single trial; omission of individual studies did not change the overall direction of effect, and the main conclusions remained consistent across outcomes (**Supplementary Fig. 15**).

Visual inspection of funnel plots for angiographic outcomes showed no obvious or consistent asymmetry for LLL and MLD, suggesting no strong evidence of small-study effects (**Supplementary Fig. 16A,B**). For DS, the funnel plot appeared less symmetric, with a small number of imprecise studies distributed toward the margins. This pattern may reflect limited study numbers and between-study variability rather than true publication bias; therefore, any inference regarding small-study effects for DS should be interpreted cautiously (**Supplementary Fig. 16C**).

## 4. Discussion

In this comprehensive systematic review and meta-analysis, we evaluated the 1-year efficacy and safety of BVS in comparison with contemporary DES in patients with CAD, while also examining mid- and long-term clinical and angiographic outcomes. The principal finding was that BVS was associated with an increased risk of ischemic events at 1 year, most notably MI, TLR, and thrombosis. Importantly, this unfavorable pattern remained evident over longer follow-up durations and was not associated with any reduction in either all-cause mortality or cardiac mortality at any assessed time point.

The slight increase in the 1-year composite clinical endpoint associated with BVS is in line with findings from RCTs and large registry-based studies. During early follow-up, both the ABSORB II trial and the AIDA trial showed higher rates of MI and scaffold thrombosis in patients receiving PLLA-based BVS compared with those treated with contemporary DES, thereby raising concerns about the routine clinical application of these devices [26,27]. Similar findings were reported in all-comer registries, indicating that the excess risk was not confined to selected trial populations [85]. The minimal between-study heterogeneity observed in the present analysis further supports the consistency and stability of this association.

Early ischemic complications after PCI have important prognostic implications. Periprocedural and early post-procedural MI are associated with impaired left ventricular function and increased long-term mortality, even when subsequent event rates appear to converge [86]. In this context, the higher rates of early MI and scaffold thrombosis observed with BVS implantation are likely to influence subsequent outcomes, thereby limiting the possibility that any delayed benefit related to scaffold resorption could offset the early harm.

Trial sequential analysis provided additional insight into the strength of evidence for the 1-year composite clinical endpoint. Although the cumulative Z-curve consistently favored DES, the final Z value did not cross the conventional significance threshold or the trial sequential monitoring boundaries. This finding indicates that the observed association is directionally consistent but remains susceptible to residual random error.

Heterogeneity in the definition of the primary composite endpoint remains an important consideration when interpreting the present findings. To address this issue, we prespecified a hierarchical endpoint extraction strategy and performed additional analyses according to both endpoint type and study design. Although the pooled estimates differed numerically across DOCE and POCE categories, the test for subgroup differences was not statistically significant, suggesting that the overall direction of effect was not materially altered by variation in endpoint composition. Likewise, the effect estimates were directionally similar across randomized controlled trials and cohort studies, and no significant interaction by study design was observed. Overall, these findings reinforce the robustness of the primary analysis, while also suggesting that the results should be interpreted with due caution in consideration of the TSA findings.

When follow-up extended beyond the first year, the clinical disadvantage associated with BVS did not diminish. Patients treated with BVS continued to experience higher rates of MI and repeat revascularization during mid- and long-term follow-up, while mortality outcomes remained similar between groups. The original premise of BVS was that removal of a permanent metallic implant might improve late outcomes after complete scaffold resorption. The available clinical data have not demonstrated the anticipated late clinical benefit of complete scaffold resorption. Instead, early device-related ischemic events appear to exert a lasting influence on subsequent clinical outcomes.

Angiographic findings provide mechanistic insight into these clinical observations. In the present analysis, BVS were associated with less favorable angiographic performance, consistent with prior intravascular imaging and angiographic studies. Thicker strut design and delayed scaffold integration have been linked to increased late lumen loss, impaired vessel healing, and disturbed flow patterns, all of which are associated with ischemic events and repeat revascularization [16,87,88,89]. Optical coherence tomography studies have shown delayed endothelial coverage and higher rates of malapposition with early-generation PLLA-based BVS than with metallic DES [90]. Although angiographic measures are not clinical endpoints, their directional consistency with the increased risks of MI and TLR may suggest a potential mechanistic link between device design and adverse clinical outcomes. However, this interpretation should remain cautious given the substantial heterogeneity observed across angiographic analyses.

The adverse outcomes observed with BVS are likely attributable primarily to the limitations of first-generation PLLA-based platforms, particularly Absorb. Although early single-arm studies reported low 1-year event rates [17], subsequent randomized trials and registry studies demonstrated substantially higher rates of device thrombosis and MI than contemporary DES [18,19], ultimately leading to the withdrawal of the Absorb scaffold from clinical use. From a structural standpoint, the lower tensile and radial strength of PLLA relative to metallic alloys necessitates thicker struts and greater vessel surface coverage to achieve adequate radial support [16]. These geometric features may alter local coronary flow patterns, delay endothelial coverage of the scaffold surface, and promote platelet adhesion and activation during the early post-implantation period [88,89]. Progressive loss of mechanical integrity during scaffold degradation may further contribute to late malapposition and persistent flow disturbances. In analyses stratified by lesion complexity, the relative risk associated with BVS appeared higher in complex or special lesions and less evident in non-complex lesions. No statistically significant interaction was observed. Prior reports have noted that fully bioresorbable scaffolds have less favorable deliverability and crossing profiles in tortuous, calcified, and bifurcation lesions than contemporary DES, and that deployment in such lesion subsets often requires more aggressive preparation and is associated with greater technical complexity [11,85,91]. Consistent with these observations, complex coronary anatomy, such as long lesions, bifurcations, severe calcification, or small and tortuous vessels, places greater technical demands on scaffold delivery, expansion, and apposition, which may accentuate the known structural and flow-related limitations of early-generation BVS rather than represent true effect modification. Subgroup analyses according to clinical presentation further showed broadly consistent effect estimates across ACS, non-ACS, and mixed populations, indicating that the early excess ischemic risk associated with BVS was not confined to a specific clinical syndrome.

Although the excess ischemic risk observed with BVS in the present analysis is most plausibly related to device- and implantation-related factors, post-PCI outcomes may also be influenced by treatment- and patient-level variables that were not uniformly reported across the included studies and therefore could not be fully addressed in this study-level meta-analysis. Differences in antithrombotic strategies, including the choice, intensity, and duration of antiplatelet therapy, may be particularly relevant in patients undergoing complex PCI, as insufficient platelet inhibition or premature discontinuation of therapy has been associated with an increased risk of thrombotic complications, whereas more intensive or prolonged regimens may increase bleeding risk [5,7,92]. Likewise, baseline comorbidity burden and overall clinical vulnerability may further modify both ischemic and bleeding risks after coronary intervention [5,7,86]. These considerations may be especially pertinent when interpreting outcomes after BVS implantation, given the recognized importance of implantation technique, vascular healing, and early scaffold-related event patterns [85,89]. In addition, emerging evidence from newer-generation scaffold platforms suggests that device performance may interact with procedural optimization and post-PCI medical management, potentially influencing longer-term outcomes [16,22,93,94,95]. Because these factors were inconsistently reported and were not available at the individual-patient level, the present analysis cannot determine to what extent the observed excess risk with BVS reflects device properties alone or their interaction with clinical management and patient vulnerability.

Material-specific differences among resorbable scaffold platforms are also clinically relevant. In subgroup analyses, the adverse clinical signal was driven primarily by PLLA-based BVS, whereas magnesium-based scaffolds did not show a statistically significant increase in ischemic risk. Preclinical studies have shown that magnesium-based scaffolds such as Magmaris achieve more rapid endothelialization than first-generation PLLA-based devices while maintaining luminal dimensions comparable to those of metallic everolimus-eluting stents at corresponding time points [96]. Experimental studies have also demonstrated lower platelet adhesion and aggregation with magnesium-based scaffolds than with Absorb, with levels comparable to or lower than those observed with newer-generation DES [97]. These findings are directionally consistent with the absence of a clear excess ischemic signal for magnesium-based scaffolds in the present analysis. However, the available comparative evidence remains limited, and no firm conclusion can yet be drawn regarding comparative safety or efficacy relative to contemporary DES.

Real-world evidence provides partial support for this observation. In the BIOSOLVE-IV registry, magnesium-based scaffolds were associated with low rates of target lesion failure and scaffold thrombosis at 24 months, with thrombotic events occurring predominantly early after implantation, often in the context of premature discontinuation of antiplatelet therapy [94,98]. However, given the non-randomized design and the lack of a concurrent DES comparator, these findings should be interpreted as supportive rather than definitive.

Data on iron-based bioresorbable scaffolds further highlight the heterogeneity across platforms. A recent randomized study comparing an iron-based sirolimus-eluting scaffold with a cobalt–chromium everolimus-eluting stent reported noninferiority for selected angiographic and clinical endpoints at 2 years, with low rates of device thrombosis [28]. Higher rates of repeat revascularization were observed, partly driven by non–ischemia-driven TLR [28]. Given the limited follow-up and the preprint status of the study, these findings require confirmation in adequately powered, peer-reviewed trials.

From the standpoint of routine clinical practice, the current evidence continues to favor contemporary DES as the standard revascularization option for most patients with CAD [5,7]. Although BVS offers theoretical benefits, such as temporary vessel support and the potential recovery of normal vascular function, the existing data do not justify its routine adoption, especially given the consistently elevated risks of MI, thrombosis, and repeat revascularization reported with early-generation devices [18,19,26,27,89]. At the same time, recent studies suggest that outcomes with newer-generation scaffolds, especially magnesium-based devices, may be more favorable than those historically observed with first-generation PLLA-based platforms. However, the available evidence remains limited and insufficient to justify a change in routine clinical practice [16,22,93,94]. In practical terms, if BVS are considered, their use should be restricted to carefully selected patients and lesions, with meticulous lesion preparation, accurate sizing, and optimized implantation technique, ideally supported by intracoronary imaging guidance where appropriate [11,85,91]. In addition, strict adherence to guideline-directed medical therapy, particularly antiplatelet treatment, remains essential to mitigate thrombotic risk [5,7,92]. Overall, the current clinical relevance of these findings lies primarily in reinforcing a cautious and selective approach to BVS rather than supporting their broad adoption in routine PCI practice.

Taken together, the currently available evidence continues to favor contemporary DES as the preferred revascularization strategy in routine practice, whereas BVS should remain investigational or be reserved for carefully selected scenarios pending further high-quality randomized evidence.

## 5. Limitations

Several limitations of the present study should be acknowledged. First, although a substantial number of randomized trials were included, part of the evidence was derived from observational studies, which are inherently susceptible to residual confounding despite careful study selection and sensitivity analyses. In addition, detailed patient-level data on antithrombotic regimens, treatment adherence, comorbidity severity, and potential drug–drug interactions were not uniformly available across the included studies. Therefore, residual confounding related to post-PCI medical management and baseline clinical vulnerability cannot be excluded. Second, heterogeneity in endpoint definitions across studies, particularly for composite outcomes such as MACE, DOCE, and POCE, may have contributed to variability in absolute event rates. However, consistent effect directions across sensitivity analyses based on endpoint definition, together with directionally concordant findings in the RCT-only analysis, mitigate this concern to some extent. Third, the included studies encompassed a wide range of scaffold platforms, implantation techniques, and eras of clinical practice. Earlier trials were conducted during the initial learning phase of bioresorbable scaffold implantation and before the widespread adoption of optimized implantation protocols, which may have influenced early clinical outcomes. Fourth, the number of studies and events involving newer-generation platforms, particularly magnesium-based and iron-based scaffolds, was limited, resulting in wide confidence intervals and reduced statistical power for subgroup analyses. Consequently, material-specific findings should be interpreted as exploratory rather than definitive. Finally, trial sequential analysis indicated that, despite an adequate accumulated information size, the available evidence for the 1-year composite clinical endpoint remains susceptible to random error. Therefore, the present findings should be interpreted in the context of the overall body of evidence rather than as conclusive proof of effect. Ongoing and future large-scale randomized trials with longer follow-up are needed to further clarify the role of bioresorbable scaffolds in contemporary coronary intervention.

## 6. Conclusion

In this systematic review and meta-analysis, bioresorbable vascular scaffolds were associated with a less favorable ischemic risk profile than contemporary drug-eluting stents, particularly with respect to MI, TLR, and device thrombosis, without evidence of a corresponding reduction in all-cause or cardiac mortality. This pattern was evident at 1 year and persisted during mid- and long-term follow-up. However, because trial sequential analysis did not demonstrate conclusive evidence for the primary endpoint, these findings should be interpreted cautiously. Overall, the currently available evidence continues to favor contemporary drug-eluting stents as the preferred revascularization strategy, whereas bioresorbable vascular scaffolds should remain investigational or be reserved for carefully selected scenarios pending further high-quality randomized evidence.

## Data Availability

The data that support the findings of this study are available from the corresponding authors upon reasonable request.
