# Supplementary material for: Efficacy and Safety of Bioresorbable Vascular Scaffolds Versus Contemporary Drug-Eluting Stents in Coronary Artery Disease: A Systematic Review, Meta-Analysis, and Trial Sequential Analysis
Source: Rev Cardiovasc Med. 2026 Oct 20;27(10):51636. doi: 10.31083/RCM51636 (PMC13732709; doi:10.31083/RCM51636)
Supplement: Supplementary file 1 [file 2153-8174-27-10-51636-s1.zip › Supplementary Material.docx]

Supplementary Table 1. Full search strategy and search terms

| **Database** | **Search strategy** | **Results** |
| --- | --- | --- |
| Pubmed | 1．(bioresorbable scaffold*[Title/Abstract] OR bioabsorbable scaffold*[Title/Abstract] OR biodegradable scaffold*[Title/Abstract] OR "bioresorbable vascular scaffold*"[Title/Abstract] OR BVS[Title/Abstract] OR Absorb[Title/Abstract] OR Magmaris[Title/Abstract] OR DREAMS[Title/Abstract] OR magnesium scaffold*[Title/Abstract] OR magnesium stent*[Title/Abstract]) 2．("coronary artery disease"[MeSH Terms] OR "coronary artery disease"[Title/Abstract] OR "coronary heart disease"[Title/Abstract] OR "ischemic heart disease"[Title/Abstract] OR "percutaneous coronary intervention"[Title/Abstract] OR PCI[Title/Abstract] 3．("drug eluting stent*"[Title/Abstract] OR DES[Title/Abstract] OR everolimus-eluting stent*[Title/Abstract] OR sirolimus-eluting stent*[Title/Abstract] OR zotarolimus-eluting stent*[Title/Abstract]) 4．(Review[Publication Type] OR Systematic Review[Publication Type] OR Meta-Analysis[Publication Type] OR Case Reports[Publication Type] OR Editorial[Publication Type] OR Letter[Publication Type] 5．1 AND 2 AND 3 NOT 4 | 266 |
| Embase | 1. (bioresorbable scaffold*:ti,ab OR bioabsorbable scaffold*:ti,ab OR biodegradable scaffold*:ti,ab OR 'bioresorbable vascular scaffold*':ti,ab OR BVS:ti,ab OR Absorb:ti,ab OR Magmaris:ti,ab OR DREAMS:ti,ab OR magnesium scaffold*:ti,ab OR magnesium stent*:ti,ab) 2. ('coronary artery disease'/exp OR 'coronary artery disease':ti,ab OR 'coronary heart disease':ti,ab OR 'ischemic heart disease':ti,ab OR 'percutaneous coronary intervention':ti,ab OR PCI:ti,ab) 3. ('drug eluting stent'/exp OR 'drug eluting stent*':ti,ab OR DES:ti,ab OR everolimus-eluting stent*:ti,ab OR sirolimus-eluting stent*:ti,ab OR zotarolimus-eluting stent*:ti,ab) 4. ('review'/it OR 'systematic review'/it OR 'meta analysis'/it OR 'case report'/it OR 'editorial'/it OR 'letter'/it OR 'conference abstract'/it OR 'conference paper'/it) 5. 1 AND 2 AND 3 NOT 4 | 583 |
| WOS | 1. TS=(bioresorbable scaffold* OR bioabsorbable scaffold* OR biodegradable scaffold* OR "bioresorbable vascular scaffold*" OR BVS OR Absorb OR Magmaris OR DREAMS OR magnesium scaffold* OR magnesium stent*) 2. TS=("coronary artery disease" OR "coronary heart disease" OR "ischemic heart disease" OR "percutaneous coronary intervention" OR PCI) 3. TS=("drug eluting stent*" OR DES OR everolimus-eluting stent* OR sirolimus-eluting stent* OR zotarolimus-eluting stent*) 4. TS=(review OR meta-analysis OR systematic review OR case report* OR editorial OR letter OR conference) 5. 1 AND 2 AND 3 NOT 4 | 496 |
| Cochrane Library | 1. (bioresorbable scaffold* OR bioabsorbable scaffold* OR biodegradable scaffold* OR "bioresorbable vascular scaffold*" OR BVS OR Absorb OR Magmaris OR DREAMS) 2. ("coronary artery disease" OR "coronary heart disease" OR "ischemic heart disease" OR "percutaneous coronary intervention" OR PCI) 3. ("drug eluting stent*" OR DES OR everolimus-eluting stent* OR sirolimus-eluting stent* OR zotarolimus-eluting stent*) 4. 1 AND 2 AND 3 | 238 |

Search was conducted to identify articles up to December 16, 2025

Supplementary Table 2. Baseline demographics and cardiovascular risk factors of included studies.

| **First Author/Study** | **Year** | **Device** | **Age(y)** | **Male (%)** | **BMI(kg/m²)** | **Hypertension (%)** | **Diabetes (%)** | **Hyperlipemia(%)** | **Smoking (%)** |
| --- | --- | --- | --- | --- | --- | --- | --- | --- | --- |
| Tommaso Gori | 2013 | BVS | 61.7±12.5 | 73 |  | 63 | 15 | 30 | 57 |
|  |  | DES | 62.0±10.9 | 70 |  | 70 | 20 | 49 | 51 |
| BVS-EXAMINATION | 2015 | BVS | 56.01 ± 12.75 | 81.4 |  | 49.7 | 12.8 | 41.7 | 61 |
|  |  | DES | 57.57 ± 12.01 | 79.7 |  | 43.8 | 12.8 | 45.5 | 75.9 |
| Bernardo Cortese | 2015 | BVS | 54 (48-60) | 74.6 |  | 48.4 | 9.8 | 73.8 | 49.2 |
|  |  | DES | 61 (53-68) | 78.7 |  | 60.8 | 19.7 | 54.4 | 43.8 |
| Charis Costopoulos | 2015 | BVS | 64.2±11.8 | 89.1 |  | 65.2 | 29.3 | 59.1 | 16.3(current smoker) |
|  |  | DES | 62.5±9.9 | 84.8 |  | 67.4 | 26.1 | 70.7 | 13 |
| ABSORB III | 2015 | BVS | 63.5±10.6 | 70.7 | 30.6±6.2 | 84.9 | 31.5 | 86.2 | 21.3(current smoker) |
|  |  | DES | 63.6±10.3 | 70.1 | 30.5±6.3 | 85 | 32.7 | 86.3 | 20.7(current smoker) |
| ABSORB China | 2015 | BVS | 57.2 ± 11.4 | 71.8 | 25.2 ± 3.4 | 58.8 | 25.2 | 42.4 | 32.8(current smoker) |
|  |  | DES | 57.6 ± 9.6 | 72.6 | 25.3 ± 3.2 | 60.3 | 23.2 | 38.4 | 35.4(current smoker) |
| Hiroyoshi Kawamoto | 2015 | BVS | 63.9 ± 11.3 | 89.8 |  | 61.9 | 27.2 | 59.9 | 51.7 |
|  |  | DES | 64.4 ± 10.6 | 84.1 |  | 62.2 | 23.3 | 63.1 | 44.9 |
| Hiroyoshi Kawamoto | 2015 | BVS | 67.1+9.4 | 78.9 | 24.0+3.0 | 78.2 | 36.1 | 82.0 | 19.9(current smoker) |
|  |  | DES | 67.3+9.6 | 73.9 | 24.3+3.0 | 79.9 | 35.8 | 82.1 | 21.6(current smoker) |
| ABSORB II | 2015 | BVS | 61.5±10 | 76 | 27.9 ±4.1 | 69 | 24 | 75 | 24(current smoker) |
|  |  | DES | 60.9±10 | 80 | 28.1±3.7 | 72 | 24 | 80 | 22(current smoker) |
| BONITO | 2016 | BVS | 60.0±9.3 | 89.5 | 28.4±5.0 | 65.4 |  |  | 24.8(current smoker) |
|  |  | DES | 63.6±10.3 | 88.8 | 28.3±4.0 | 69 |  |  | 20.6(current smoker) |
| Daniel Dalos | 2016 | BVS | 54.0 ± 11.2 | 73 |  | 58 | 31 | 65 | 34(current smoker) |
|  |  | DES | 61.7 ± 11.4 | 88 |  | 74 | 26 | 62 | 36(current smoker) |
| José de Ribamar Costa Jr1 | 2016 | BVS | 61.1±10.7 | 74.3 |  | 71.4 | 26.5 | 71.9 | 23.2 |
|  |  | DES | 61.4±10.3 | 72.7 |  | 75 | 26.9 | 75.4 | 23.3 |
| Jiang Ming Fam | 2016 | BVS | 56.31±10.22 | 72.2 |  | 39.7 | 11.3 | 28.4 | 41 |
|  |  | DES | 54.90±11.52 | 74.8 |  | 37.1 | 9.9 | 27.1 | 58.9 |
| Yoichi Imori | 2016 | BVS | 59.7 ± 13.0 | 79.4 |  | 56.1 | 14 | 41.1 | 51.4 |
|  |  | DES | 61.5 ± 11.9 | 80.5 |  | 54.4 | 16.7 | 42.8 | 41.4 |
| UNDERDOGS | 2016 | BVS | 58 ± 10 | 88 |  | 62 | 28 | 59 | 37(current smoker) |
|  |  | DES | 59 ± 7 | 83 |  | 64 | 25 | 57 | 39(current smoker) |
| V. F. Panoulas | 2016 | BVS | 64.5±10.3 | 91.4 |  | 65.7 | 31.4 | 70 | 57.1 |
|  |  | DES | 66.0±9.7 | 95.7 |  | 50.7 | 31.4 | 54.3 | 47.8 |
| TROFI II | 2016 | BVS | 59.1 ± 10.7 | 76.8 | 27.0 ± 4.1 | 44.1 | 18.9 | 63.8 | 48.4(current smoker) |
|  |  | DES | 58.2 ± 9.6 | 87.5 | 27.7 ± 4.2 | 36.5 | 14.7 | 57.3 | 49.5(current smoker) |
| Corrado Tamburino | 2016 | BVS | 62.75 ± 10.65 | 77.9 |  | 78.9 | 27.5 | 67.2 | 28.4(current smoker) |
|  |  | DES | 63.04 ± 11.02 | 77.9 |  | 78.9 | 27.1 | 67.8 | 27.8(current smoker) |
| Toru Naganuma | 2017 | BVS | 62.7 ± 10.6 | 89.8 |  | 64.6 | 31.3 | 63.3 | 12.9(current smoker) |
|  |  | DES | 64.1 ± 9.7 | 83.5 |  | 73.9 | 26.1 | 75.6 | 31.8(current smoker) |
| AIDA | 2017 | BVS | 64.3±10.6 | 72.5 |  | 50.9 | 18.5 | 37.6 | 28.6(current smoker) |
|  |  | DES | 64.0±10.5 | 76.0 |  | 50.5 | 16.6 | 38.3 | 31.7(current smoker) |
| Carlos M. Campos | 2017 | BVS | 61.5 ± 10.3 | 72.1 |  | 81.9 | 17.2 | 76.7 | 23.7(current smoker) |
|  |  | DES | 61.2 ± 9.6 | 74.2 |  | 87.1 | 19.4 | 79.7 | 15.9(current smoker) |
| Yaling Han | 2018 | BVS | 58.5 ± 8.8 | 67.6 | 24.9 ± 3.3 | 54.3 | 19.1 | 19.4 | 24.5(current smoker) |
|  |  | DES | 58.9 ± 8.7 | 68.1 | 24.5 ± 3.1 | 52.8 | 19.9 | 16.7 | 30.5(current smoker) |
| Jose M. de la Torre Hernandez | 2016 | BVS | 60.8±11 | 79 |  | 56 | 16 | 58 | 21 |
|  |  | DES | 61.3±12 | 76 |  | 61 | 20 | 62 | 19 |
| Andrea Cuculo | 2016 | BVS | 54.85±7.76 | 85 |  | 67 | 25 | 75 |  |
|  |  | DES | 66.62±10.88 | 80 |  | 69 | 36 | 64 |  |
| EVERBIO II | 2016 | BVS | 65±11 | 78 |  | 55 | 22 | 56 | 36 |
|  |  | DP-DES | 65±11 | 80 |  | 64 | 16 | 63 | 38 |
|  |  | BP-DES | 65±10 | 80 |  | 63 | 33 | 65 | 31 |
| Kugler, Christine | 2017 | BVS | 60.5 ± 7.8 | 85.7 | 27.8 ± 3.7 | 64.3 | 14.3 | 71.4 | 57.1 |
|  |  | DES | 65.7 ± 9.4 | 93.3 | 28.3 ± 3.0 | 80 | 13.3 | 73.3 | 66.7 |
| Frédéric Maes | 2018 | BVS | 59 ± 11 | 83 | 30 ± 5 | 57 | 25 | 75 | 28 |
|  |  | DES | 59 ± 10 | 83 | 29 ± 5 | 59 | 25 | 74 | 32 |
| BIORESOLVE-ISR | 2018 | BVS | 65.8 ± 10.2 | 82 | 27.0 ± 4.1 | 68.5 | 33.7 | 70.7 | 46.7 |
|  |  | DES | 66.5 ± 10.4 | 83 | 28.0 ± 3.9 | 76.1 | 31.5 | 71.4 | 44.6 |
| ABSORB IV | 2018 | BVS | 63.1±10.1 | 72 | 30.3±5.9 | 79 | 32 | 80 | 22(current smoker) |
|  |  | DES | 62.2±10.3 | 72 | 30.2±6.1 | 79 | 32 | 79 | 23(current smoker) |
| Giuseppe Tarantini | 2018 | BVS | 64.4 ± 9.5 | 77.2 |  | 61.4 | 26.1 | 61.5 | 54.9 |
|  |  | DES | 64.9 ± 9.8 | 75.1 |  | 56.4 | 26.4 | 59.1 | 57 |
| ISAR-Absorb MI | 2018 | BVS | 61.7 ± 11.0 | 79.8 | 27.0 ± 4. | 53.5 | 21.6 | 43.5 | 44.5 |
|  |  | DES | 63.3 ± 9.9 | 73 | 27.0 ± 3.7 | 62.1 | 19.3 | 47.6 | 43.2 |
| COVER-AMI | 2019 | BVS | 56.5 ± 13.6 | 90 |  | 30 | 10 | 20 | 50 |
|  |  | DES | 61.4 ± 9.0 | 70 |  | 30 | 0 | 0 | 40 |
| Yizhe Wu | 2019 | BVS | 60.2 ± 8.3 | 67.5 | 24.6 ± 3.2 | 60.5 | 24.5 | 14.5 | 27(current smoker) |
|  |  | DES | 60.0 ± 8.6 | 67.2 | 25.1 ± 3.3 | 53.8 | 21.5 | 12.3 | 28.7(current smoker) |
| Piotr Desperak | 2020 | BVS | 53.8 ±10.1 | 78.9 |  | 56.1 | 21.9 | 68.3 | 37.4 |
|  |  | DES | 61.0 ±12.2 | 78 |  | 65.2 | 21.3 | 53.9 | 43.3 |
| Alexandre Hideo-Kajita | 2020 | BVS | 65.5±10.8 | 63.6 |  | 79.4 | 25 | 62 | 55.4 |
|  |  | DES | 62.7±10.4 | 78.2 |  | 77.8 | 29 | 69.9 | 68.3 |
| Yongcheol Kim | 2020 | BVS | 54.8 ± 10.8 | 89.5 | 24.5 ± 3.1 | 32.6 | 18.9 | 10.5 | 53.7(current smoker) |
|  |  | DES | 56.2 ± 10.3 | 87.3 | 24.3 ± 2.9 | 37.1 | 22.4 | 8.8 | 54.6(current smoker) |
| Jongkwon Seo | 2020 | BVS | 63 ± 10 | 75 |  | 52 | 31 | 81 | 19(current smoker) |
|  |  | DES | 62 ± 10 | 81 |  | 58 | 31 | 85 | 19(current smoker) |
| COMPARE-ABSORB |  | BVS | 62 (56-69) | 79.5 | 27 (25-31) | 71.6 | 34.6 | 66.3 | 28.8(current smoker) |
|  |  | DES | 63 (56-69) | 76.3 | 27 (25-30) | 69.2 | 36.1 | 66.3 | 26.9(current smoker) |
| Andrew Kei-Yan Ng | 2021 | BVS | 60.4 ± 10.7 | 79.4 |  | 48.8 | 28.2 | 65.3 | 35.9 |
|  |  | DES | 64.7 ± 10.7 | 73 |  | 62.1 | 38.9 | 45.9 | 31.6 |
| FUTURE-II | 2021 | BVS | 56.5 ± 9.6 | 72.1 | 25.3 ± 3.29 | 54.9 | 25.6 | 40.9 | 38.6(current smoker) |
|  |  | DES | 57.4 ± 9.5 | 77.1 | 25.2 ± 3.11 | 60.6 | 29.8 | 41.7 | 36.2(current smoker) |
| Quentin de Hemptinne | 2022 | BVS | 54.7 ± 10.2 | 76.7 |  | 53.3 | 16.7 | 60 | 76.7 |
|  |  | BP-DES | 59.2 ± 12.0 | 73.3 |  | 53.3 | 13.3 | 56.7 | 83.3 |
|  |  | DP-DES | 62.6 ± 9.4 | 70 |  | 63.3 | 20 | 63.3 | 70 |
| PRAGUE-22 | 2022 | BVS | 57.0 ± 10.5 | 64 | 28.5 ± 4.3 |  | 12 |  | 72 |
|  |  | DES | 55.5 ± 9.2 | 76 | 29.7 ± 4.6 |  | 32 |  | 56 |
| Piotr Rola | 2022 | Magmaris | 66.3 ± 8.9 | 77.7 |  | 88.6 | 37.3 | 78.7 |  |
|  |  | BP-DES | 65.2 ± 9.34 | 75.7 |  | 93.5 | 34.9 | 76.9 |  |
| Eriksen, Erlend | 2023 | BVS | 60.7 ± 10.5 | 77.4 | 28.6 ± 1.2 | 29(Antihypertensive treatment) | 9.7 | 22.6(Statin treatment) | 67.7 |
|  |  | DES | 62.3 ± 10.6 | 71.4 | 27.6 ± 1.2 | 28.6 | 6.7 | 14.3 | 77.1 |
| Joo Myung Lee | 2023 | BVS | 56.7 ± 10.3 | 82.8 |  | 44.9 | 26.7 | 49.5 | 29.8(current smoker) |
|  |  | DES | 63.3 ± 10.3 | 80.5 |  | 61.3 | 45.2 | 52.7 | 28.5 |
| RIBS VI | 2024 | BVS | 65 ± 10 | 87 |  | 77 | 49 | 80 | 67 |
|  |  | DCB | 67 ± 10 | 84 |  | 72 | 42 | 72 | 42 |
|  |  | DES | 65 ± 10 | 85 |  | 76 | 34 | 74 | 37 |
| ISAR-RESORB | 2024 | BVS | 68.5 ± 10.5 | 71.9 |  | 86 | 28.1 | 78.9 | 45.6 |
|  |  | BP-DES | 66.7 ± 11.4 | 78.3 |  | 91.7 | 36.7 | 85 | 45 |
| Adrian Włodarczak | 2024 | BVS | 66.3 ±8.9 | 77.7 |  | 88.6 | 37.3 | 78.7 |  |
|  |  | BP-DES | 65.2 ±9.3 | 75.7 |  | 93.5 | 34.9 | 76.9 |  |
| Jun Li | 2025 | BVS | 62 ± 11 | 73 |  |  | 16 | 31 | 44 |
|  |  | BP-DES | 64 ± 6 | 92 |  |  | 18 | 34 | 25 |
| BIOHEART-II | 2025 | BVS | 58.8 ± 9.6 | 75.2 | 25.8 ± 3.4 | 61.5 | 23.9 | 47.7 | 50.9 |
|  |  | DES | 58.7 ± 8.7 | 72.7 | 25.1 ± 3.0 | 58.3 | 28.2 | 50.5 | 51.6 |
| IRONMAN II | 2025 | BVS | 59.4 ± 9.5 | 70.7 | 25.4 ± 3.3 | 62.9 | 27.4 | 34.4 | 27.4(current smoker) |
|  |  | DES | 60.4 ± 9.9 | 68 | 24.9 ± 3.3 | 63.7 | 29.7 | 32.4 | 24.3 |

Supplementary Table 3. Clinical history and presentation of included studies

| **FirstAuthor/Study** | **Year** | **Device** | **PriorMI (%)** | **PriorPCI (%)** | **PriorCABG(%)** | **FamilyHistory(%)** | **Renal insufficiency(%)** | **Stable angina（%）** | **Silent ischemia(%)** | **ACS(%)** | **UA(%)** | **NSTEMI(%)** | **STEMI(%)** |
| --- | --- | --- | --- | --- | --- | --- | --- | --- | --- | --- | --- | --- | --- |
| Tommaso Gori | 2013 | BVS | 10 |  |  |  |  |  |  |  | 16 | 40 | 44 |
|  |  | DES | 15 |  |  |  |  |  |  |  | 19 | 45 | 36 |
| BVS-EXAMINATION | 2015 | BVS | 3.5 | 3.4 | 1 |  |  |  |  |  |  |  |  |
|  |  | DES | 3.5 | 3.8 | 0.3 |  |  |  |  |  |  |  |  |
| Bernardo Cortese | 2015 | BVS | 12.3 | 10.7 | 0 | 40.2 |  |  |  |  |  |  |  |
|  |  | DES | 5.9 | 5.9 | 1.1 | 28.1 |  |  |  |  |  |  |  |
| Charis Costopoulos | 2015 | BVS | 28.3 | 53.3 | 9.8 | 45.7 |  | 89.1 |  | 10.9 |  |  |  |
|  |  | DES | 34.8 | 40.2 | 9.8 | 35.9 |  | 88 |  | 12 |  |  |  |
| ABSORB III | 2015 | BVS | 21.5 |  |  |  | 10.8 | 57.3 | 10 |  | 26.9 |  |  |
|  |  | DES | 22 |  |  |  | 11.1 | 60.8 | 10.2 |  | 24.5 |  |  |
| ABSORB China | 2015 | BVS | 16.8 | 9.7 |  |  |  | 22.3 | 3.8 |  | 64.7 |  |  |
|  |  | DES | 16 | 8 |  |  |  | 16.9 | 5.5 |  | 64.1 |  |  |
| Hiroyoshi Kawamoto | 2015 | BVS | 26.5 | 48.3 | 7.5 | 41.5 |  |  |  |  |  |  |  |
|  |  | DES | 33.8 | 41.2 | 10.2 | 50.6 |  |  |  |  |  |  |  |
| Hiroyoshi Kawamoto | 2015 | BVS | 16 |  |  | 6.5(premature) |  | 63.9 | 26.3 |  | 9.8 |  |  |
|  |  | DES | 23.9 |  |  | 8.1(premature) |  | 65.7 | 17.9 |  | 16.4 |  |  |
| ABSORB II | 2015 | BVS | 28 | 12 |  | 37 |  | 64 | 13 |  | 20 |  |  |
|  |  | DES | 29 | 9 |  | 41 |  | 64 | 11 |  | 22 |  |  |
| BONITO | 2016 | BVS | 32.7 | 43.8 | 2.6 | 29.6 | 5.5 |  | 35.9 | 9.7 |  |  |  |
|  |  | DES | 41.9 | 56.3 | 9.9 | 36.3 | 15.9 |  | 31.5 | 12 |  |  |  |
| Daniel Dalos | 2016 | BVS |  |  |  | 23 |  |  |  | 39 |  |  |  |
|  |  | DES |  |  |  | 29 |  |  |  | 21 |  |  |  |
| José de Ribamar Costa Jr | 2016 | BVS |  | 27.6 | 1.7 | 40 |  | 56.8 |  |  | 26.5 | 16.7 |  |
|  |  | DES |  | 23.6 | 1.9 | 35.5 |  | 59.3 |  |  | 24.6 | 16.1 |  |
| Jiang Ming Fam | 2016 | BVS |  |  |  | 33.8 |  |  |  |  |  |  |  |
|  |  | DES |  |  |  | 34.4 |  |  |  |  |  |  |  |
| Yoichi Imori | 2016 | BVS |  |  |  |  |  |  |  |  |  |  | 49.1 |
|  |  | DES |  |  |  |  |  |  |  |  |  |  | 45.1 |
| UNDERDOGS | 2016 | BVS | 20 | 9 | 2 | 27 |  | 46 |  |  | 20 | 23 | 11 |
|  |  | DES | 22 | 10 | 2 | 31 |  | 52 |  |  | 17 | 21 | 11 |
| V. F. Panoulas | 2016 | BVS | 28.3 | 48.6 | 7.1 | 41.4 |  |  |  |  |  |  |  |
|  |  | DES | 41.4 | 50 | 11.6 | 40 |  |  |  |  |  |  |  |
| TROFI II | 2016 | BVS | 2.1 | 4.2 |  |  |  |  |  |  |  |  |  |
|  |  | DES | 3.1 | 3.1 |  |  |  |  |  |  |  |  |  |
| Corrado Tamburino | 2016 | BVS |  | 35.5 | 5.9 | 37.3 | 16 |  |  | 42.3 |  | 15.5 | 10.7 |
|  |  | DES |  | 35.3 | 5.1 | 37.7 | 19.3 |  |  | 42.5 |  | 16.7 | 10.5 |
| Toru Naganuma | 2017 | BVS |  | 42.9 |  | 36.7 |  |  |  | 10.9 | 9.5 | 0 | 1.4 |
|  |  | DES |  | 50.6 |  | 36.4 |  |  |  | 10.8 | 8.5 | 2.3 | 0 |
| AIDA | 2017 | BVS | 18 | 21.9 | 4.1 | 50.9 | 7.6 | 39.1 |  |  | 7.6 | 20 | 26 |
|  |  | DES | 18.7 | 20.0 | 2.8 | 52.9 | 9.9 | 40.2 |  |  | 9.4 | 20.8 | 24.4 |
| Carlos M. Campos | 2017 | BVS | 31.5 | 34.0 | 2.8 | 38.0 |  |  |  |  | 27.0 |  |  |
|  |  | DES | 26.1 | 26.5 | 1.9 | 45.5 |  |  |  |  | 27.1 |  |  |
| Yaling Han | 2018 | BVS | 5.4 | 5 | 0 |  | 0.7 | 16.5 | 2.2 |  | 79.1 | 0.4 | 1.8 |
|  |  | DES | 7.1 | 3.9 | 0.4 |  | 0.7 | 15.2 | 1.4 |  | 79.8 | 0.4 | 3.2 |
| Jose M. de la Torre Hernandez | 2016 | BVS | 18 | 21 | 1 | 87 | 5 |  |  |  | 55 |  |  |
|  |  | DES | 22 | 26 | 1 | 83 | 4 |  |  |  | 51 |  |  |
| Andrea Cuculo | 2016 | BVS |  |  |  |  |  | 33 |  |  | 16 | 35 | 16 |
|  |  | DES |  |  |  |  |  | 25 |  |  | 18 | 35 | 22 |
| EVERBIO II | 2016 | BVS | 14 | 32 | 8 | 30 |  | 53 | 12 |  | 8 | 17 | 12 |
|  |  | DP-DES | 18 | 31 | 14 | 29 |  | 59 | 8 |  | 6 | 20 | 8 |
|  |  | BP-DES | 20 | 29 | 20 | 29 |  | 34 | 19 |  | 11 | 26 | 10 |
| Kugler, Christine | 2017 | BVS |  |  | 0 |  | 7.1 |  |  |  |  |  |  |
|  |  | DES |  |  | 13.3 |  | 6.7 |  |  |  |  |  |  |
| Frédéric Maes | 2018 | BVS | 17 | 35 | 4.2 | 34 |  | 36 |  |  | 39 | 15 | 9.3 |
|  |  | DES | 13 | 32 | 5.9 | 25 |  | 36 |  |  | 39 | 15 | 9.3 |
| BIORESOLVE-ISR | 2018 | BVS | 57.6 |  | 13 |  | 14.1 | 54.4 |  |  | 38 | | 7.6 |
|  |  | DES | 72.8 |  | 14.1 |  | 6.5 | 53.3 |  |  | 39.1 | | 7.6 |
| ABSORB IV | 2018 | BVS | 18 |  |  |  | 14 | 51 | 7 |  | 18 | 22 | 3 |
|  |  | DES | 19 |  |  |  | 13 | 51 | 8 |  | 18 | 22 | 4 |
| Giuseppe Tarantini | 2018 | BVS | 23.1 | 27.3 | |  | 1.8 |  |  | 53.4 |  |  | 14.2 |
|  |  | DES | 22.6 | 26.4 | |  | 2.7 |  |  | 54 |  |  | 14.2 |
| ISAR-Absorb MI | 2018 | BVS | 6.9 | 8.8 | 0 | 23.4 |  |  |  |  |  | 23.7 | 76.3 |
|  |  | DES | 6.7 | 7.9 | 0 | 25.3 |  |  |  |  |  | 27 | 73 |
| COVER-AMI | 2019 | BVS |  |  |  |  |  |  |  |  |  |  |  |
|  |  | DES |  |  |  |  |  |  |  |  |  |  |  |
| Yizhe Wu | 2019 | BVS |  | 13.5 | 0 |  |  | 30 |  |  | 39.5 | 14 | |
|  |  | DES |  | 8.2 | 0 |  |  | 27.7 |  |  | 39.5 | 15.4 | |
| Piotr Desperak | 2020 | BVS | 12.2 | 9.8 |  |  | 8.5 |  |  |  |  |  |  |
|  |  | DES | 17 | 9.8 |  |  | 17.7 |  |  |  |  |  |  |
| Alexandre Hideo-Kajita | 2020 | BVS | 22.8 | 41.3 |  |  |  |  |  |  | 12.5 |  |  |
|  |  | DES | 30.7 | 42.9 |  |  |  |  |  |  | 19.5 |  |  |
| Yongcheol Kim | 2020 | BVS | 2.1 |  |  | 8.4 |  |  |  |  |  | 52.6 | 47.4 |
|  |  | DES | 3.9 |  |  | 10.2 |  |  |  |  |  | 52.7 | 47.3 |
| Jongkwon Seo | 2020 | BVS |  | 16 |  |  |  | 54 |  |  | 41 | 5 | |
|  |  | DES |  | 13 |  |  |  | 56 |  |  | 37 | 7 | |
| COMPARE-ABSORB |  | BVS | 18.2 | 27 | 1.9 | 36.2 | 3.9 | 40.4 | 7.4 | 52.1 | 17.6 | 21.6 | 12.9 |
|  |  | DES | 20.2 | 29 | 2.6 | 31.7 | 6 | 42.5 | 8.9 | 48.7 | 17.2 | 19 | 12.5 |
| Andrew Kei-Yan Ng | 2021 | BVS | 4.1 | 8.8 |  |  | 1.2 | 80 |  |  |  | 20 | 0 |
|  |  | DES | 10.1 | 32.3 |  |  | 1.7 | 74.7 |  |  |  | 17.4 | 7.9 |
| FUTURE-II | 2021 | BVS | 14.4 | 14 |  | 9.6 |  | 17.7 | 9.3 |  | 73 |  |  |
|  |  | DES | 11.9 | 15.3 |  | 8.7 |  | 18.8 | 7.3 |  | 73.9 |  |  |
| Quentin de Hemptinne | 2022 | BVS | 0 | 0 |  |  | 3.3 |  |  |  |  |  |  |
|  |  | BP-DES | 3.3 | 3.3 |  |  | 10 |  |  |  |  |  |  |
|  |  | DP-DES | 3.3 | 3.3 |  |  | 10 |  |  |  |  |  |  |
| PRAGUE-22 | 2022 | BVS |  |  |  |  |  |  |  |  | 4 | 24 | 72 |
|  |  | DES |  |  |  |  |  |  |  |  | 16 | 28 | 56 |
| Piotr Rola | 2022 | BVS | 30.5 | 40.4 |  |  |  |  |  |  | 15.5 | 84.5 |  |
|  |  | BP-DES | 35.5 | 36.1 |  |  |  |  |  |  | 47.9 | 52.1 |  |
| Eriksen, Erlend | 2023 | BVS |  | 0 | 0 |  |  |  |  |  |  |  |  |
|  |  | DES |  | 2.9 | 0 |  |  |  |  |  |  |  |  |
| Joo Myung Lee | 2023 | BVS | 4 |  |  |  | 0.8 | 39 |  | 61 | 23.1 | 37.9 | |
|  |  | DES | 6.6 |  |  |  | 6.9 | 49.5 |  | 50.5 | 31 | 19.5 | |
| RIBS VI | 2024 | BVS | 61 |  | 8 |  |  | 51 | |  | 49 |  |  |
|  |  | DCB | 52 |  | 8 |  |  | 53 | |  | 47 |  |  |
|  |  | DES | 53 |  | 10 |  |  | 51 | |  | 49 |  |  |
| ISAR-RESORB | 2024 | BVS | 21.1 | 49.1 | 5.3 | 35.1 |  | 28.6 | 42.9 |  | 19.6 | 7.1 | 1.8 |
|  |  | BP-DES | 15 | 50 | 5 | 35 |  | 35 | 31.7 |  | 23.3 | 10 | 0 |
| Adrian Włodarczak | 2024 | BVS | 30.5 | 40.4 |  |  |  |  |  |  | 15.5 | 84.5 |  |
|  |  | BP-DES | 35.5 | 36.1 |  |  |  |  |  |  | 47.9 | 52.1 |  |
| Jun Li | 2025 | BVS |  |  |  | 35 |  |  |  |  |  | 73 | 27 |
|  |  | BP-DES |  |  |  | 34 |  |  |  |  |  | 76 | 24 |
| BIOHEART-II | 2025 | BVS | 13.8 | 17 | 0.5 | 10.6 |  | 17.4 | 2.3 |  | 18.8 | 0.9 | 1.4 |
|  |  | DES | 13.9 | 19.9 | 0 | 8.8 |  | 21.3 | 4.6 |  | 25 | 0.9 | 0.9 |
| IRONMAN II | 2025 | BVS | 6.9 | 18.1 |  |  |  | 19.3 | 6.2 |  | 75.3 |  |  |
|  |  | DES | 5.8 | 17.4 |  |  |  | 16.6 | 4.6 |  | 81.1 |  |  |

Supplementary Table 4.Target lesion characteristics of included studies.

| **First Author/Study** | **Year** | **Device** | **LM**  **(%)** | **LAD**  **(%)** | **RCA(%)** | **LCx(%)** | **Bifurcation(%)** | **Multivessel disease(%)** | **Calcified(%)** | **Thrombus**  **(%)** | **CTO(%)** | **In-stent restenosis**  **(%)** | **RVD**  **(mm)** |
| --- | --- | --- | --- | --- | --- | --- | --- | --- | --- | --- | --- | --- | --- |
| Tommaso Gori | 2013 | BVS |  | 42 | 38 | 19 |  | 57 |  | 84 | 48 |  |  |
|  |  | DES |  | 47 | 28 | 25 |  | 64 |  | 73 | 28 |  |  |
| BVS-EXAMINATION | 2015 | BVS | 0.3 | 50 | 39.3 | 10 |  | 8.2 |  |  |  |  |  |
|  |  | DES | 0.7 | 40.3 | 43.4 | 15.5 |  | 9.7 |  |  |  |  |  |
| Bernardo Cortese | 2015 | BVS |  | 60.7 | 26.7 | 12.6 | 3.7 | 42.6 |  |  | 40 |  |  |
|  |  | DES |  | 58.5 | 28.1 | 13.4 | 20.9 | 51.2 |  |  | 59.6 |  |  |
| Charis Costopoulos | 2015 | BVS | 0.7 | 62.8 | 13.1 | 23.4 | 45.2 | 51.1 | 20.4 |  | 7.3 | 5.8 |  |
|  |  | DES | 2.4 | 64.5 | 14.5 | 18.5 | 40.3 | 50 | 16.1 |  | 5.6 | 11.3 |  |
| ABSORB III | 2015 | BVS | 44.5 |  | 29.2 | 26.2 |  |  |  |  |  |  | 2.67±0.45 |
|  |  | DES | 42.2 |  | 27.2 | 30.6 |  |  |  |  |  |  | 2.65±0.46 |
| ABSORB China | 2015 | BVS |  | 55.4 | 25.1 | 19.5 | 50.2 | 37 | 17.5 |  |  |  | 2.81 ± 0.03 |
|  |  | DES |  | 52.4 | 23.4 | 24.2 | 48.6 | 43.5 | 15.5 |  |  |  | 2.82 ± 0.03 |
| Hiroyoshi Kawamoto | 2015 | BVS |  | 59.4 | 12.7 | 25.9 |  |  |  |  | 6.1 | 7.6 | 2.81 ± 0.51 |
|  |  | DES |  | 51.8 | 21.7 | 22.8 |  |  |  |  | 9.6 | 8.1 | 2.75 ± 0.66 |
| Hiroyoshi Kawamoto | 2015 | BVS |  | 46.2 | 30.9 | 22.9 |  | 3.4 | 27.7 |  |  |  |  |
|  |  | DES |  | 42.3 | 31.4 | 26.3 |  | 2.2 | 32.8 |  |  |  |  |
| ABSORB II | 2015 | BVS |  | 45 | 26 | 29 |  | 17 | 13 |  |  |  |  |
|  |  | DES |  | 46 | 31 | 23 |  | 15 | 15 |  |  |  |  |
| BONITO | 2016 | BVS |  | 46.4 | 34.6 | 19 |  |  | 45.8 |  |  |  | 3.0±0.4 |
|  |  | DES |  | 33.1 | 45.8 | 21.1 |  |  | 38.5 |  |  |  | 2.8±0.6 |
| Daniel Dalos | 2016 | BVS |  | 73 | 15 | 11 |  |  |  |  |  |  |  |
|  |  | DES |  | 56 | 15 | 26 |  |  |  |  |  |  |  |
| José de Ribamar Costa Jr1 | 2016 | BVS | 0.1 | 48.4 | 25.6 | 25.9 | 5.9 | 17.5 |  |  |  |  | 2.64±0.39 |
|  |  | DES | 0.1 | 42.1 | 25.6 | 32.2 | 6.4 | 20.1 |  |  |  |  | 2.69±0.44 |
| Jiang Ming Fam | 2016 | BVS |  | 42.4 | 33.8 | 21.2 |  |  |  |  |  |  |  |
|  |  | DES |  | 41.1 | 30.5 | 26.5 |  |  |  |  |  |  |  |
| Yoichi Imori | 2016 | BVS | 0.4 | 56.6 | 26.9 | 16.1 |  |  |  |  |  |  |  |
|  |  | DES | 2.4 | 45.3 | 30.7 | 17.9 |  |  |  |  |  |  |  |
| UNDERDOGS | 2016 | BVS |  | 87 | 46 | 54 |  | 59 |  |  |  |  |  |
|  |  | DES |  | 87 | 48 | 52 |  | 58 |  |  |  |  |  |
| V. F. Panoulas | 2016 | BVS | 1 | 64.4 | 12.9 | 21.8 | 45.5 | 40 | 34.7 |  | 8.9 | 5.9 |  |
|  |  | DES | 5 | 47.5 | 26.7 | 20 | 30 | 40 | 19.2 |  | 16.7 | 10 |  |
| TROFI II | 2016 | BVS |  | 35.8 | 46.3 | 17.9 |  |  |  |  |  |  |  |
|  |  | DES |  | 41.8 | 44.9 | 13.3 |  |  |  |  |  |  |  |
| Corrado Tamburino | 2016 | BVS | 1.3 |  |  |  | 22 | 57.9 |  |  | 7.7 |  | 2.97 ± 0.53 |
|  |  | DES | 1 |  |  |  | 22.5 | 59.8 |  |  | 7.5 |  | 2.95 ± 0.54 |
| Toru Naganuma | 2017 | BVS |  | 75.3 | 9.6 | 15.1 |  | 40.1 | 25.3 |  | 6.6 | 5.4 |  |
|  |  | DES |  | 59.5 | 9.2 | 31.4 |  | 45.5 | 17.8 |  | 5.9 | 11.4 |  |
| AIDA | 2017 | BVS |  |  |  |  |  |  |  |  |  |  |  |
|  |  | DES |  |  |  |  |  |  |  |  |  |  |  |
| Carlos M. Campos | 2017 | BVS |  |  |  |  |  | 19.5 |  |  |  |  | 2.64 ± 0.37 |
|  |  | DES |  |  |  |  |  | 25.8 |  |  |  |  | 2.73 ± 0.47 |
| Yaling Han | 2018 | BVS |  | 65.1 | 21.9 | 12.9 |  |  |  |  |  |  | 2.95 ± 0.45 |
|  |  | DES |  | 63.3 | 18 | 18.7 |  |  |  |  |  |  | 2.93 ± 0.47 |
| Jose M. de la Torre Hernandez | 2016 | BVS |  | 52.8 | 32 | 15 |  |  | 20 |  |  |  | 2.91±0.4 |
|  |  | DES |  | 54.6 | 28.4 | 16.9 |  |  | 23 |  |  |  | 2.89±0.4 |
| Andrea Cuculo | 2016 | BVS |  |  |  |  | 25 |  |  |  | 4 |  |  |
|  |  | DES |  |  |  |  | 27 |  |  |  | 11 |  |  |
| EVERBIO II | 2016 | BVS |  |  |  |  |  |  |  |  |  |  | 2.77 ± 0.60 |
|  |  | DP-DES |  |  |  |  |  |  |  |  |  |  | 2.39 ± 0.70 |
|  |  | BP-DES |  |  |  |  |  |  |  |  |  |  | 2.53 ± 0.84 |
| Kugler, Christine | 2017 | BVS |  | 20 | 73.3 | 6.7 |  |  | 46.7 |  |  |  |  |
|  |  | DES |  | 33.3 | 66.7 | 0 |  |  | 53.3 |  |  |  |  |
| Frédéric Maes | 2018 | BVS | 0.6 | 78 | 12 | 9.6 | 21 |  |  |  |  |  |  |
|  |  | DES | 0.9 | 27 | 45 | 28 | 19 |  |  |  |  |  |  |
| BIORESOLVE-ISR | 2018 | BVS |  | 52 | 28 | 18 |  | 40.2 |  |  |  |  | 3.0 ± 0.44 |
|  |  | DES |  | 43 | 33 | 14 |  | 72.8 |  |  |  |  | 3.0 ±0.35 |
| ABSORB IV | 2018 | BVS |  | 44 | 31 | 26 |  | 13 | 24 |  |  |  | 2.9±0.39 |
|  |  | DES |  | 44 | 30 | 26 |  | 13 | 24 |  |  |  | 2.89±0.38 |
| Giuseppe Tarantini | 2018 | BVS | 0.9 | 41 | 27.1 | 31 | 11 | 63.2 | 12.8 |  | 2.7 | 2.7 | 2.4 ± 0.2 |
|  |  | DES | 1.6 | 41.4 | 20.6 | 36.4 | 6.2 | 65.6 | 14.5 |  | 2.4 | 3.3 | 2.5 ± 0.2 |
| ISAR-Absorb MI | 2018 | BVS |  | 47.4 | 35.3 | 17.3 | 16.3 | 40.5 |  |  |  |  | 2.89 ± 0.42 |
|  |  | DES |  | 48.3 | 40.4 | 11.2 | 19.1 | 41.6 |  |  |  |  | 2.95 ± 0.43 |
| COVER-AMI | 2019 | BVS |  | 90 | 50 | 50 |  | 80 |  |  |  |  | 3.4 ± 0.5 |
|  |  | DES |  | 70 | 80 | 10 |  | 60 |  |  |  |  | 3.7 ± 0.4 |
| Yizhe Wu | 2019 | BVS |  |  |  |  |  | 5 |  |  |  |  |  |
|  |  | DES |  |  |  |  |  | 10.2 |  |  |  |  |  |
| Piotr Desperak | 2020 | BVS | 0 | 60.3 | 17.2 | 22.5 | 17.9 |  |  | 24.5 |  |  |  |
|  |  | DES | 4.5 | 50.6 | 31.2 | 12.5 | 18.2 |  |  | 25 |  |  |  |
| Alexandre Hideo-Kajita | 2020 | BVS | 0 | 41.3 | 33.9 | 24.9 | 2.6 |  |  |  |  |  | 2.7±0.4 |
|  |  | DES | 0 | 44.8 | 33 | 22.1 | 1.2 |  |  |  |  |  | 2.8±0.5 |
| Yongcheol Kim | 2020 | BVS | 0 | 57.9 | 27.4 | 14.7 |  |  |  |  |  |  |  |
|  |  | DES | 0 | 57.1 | 27.8 | 15.1 |  |  |  |  |  |  |  |
| Jongkwon Seo | 2020 | BVS |  | 57 | 28 | 14 |  |  |  |  | 14 |  | 2.95 ± 0.49 |
|  |  | DES |  | 57 | 24 | 19 |  |  |  |  | 11 |  | 2.87 ± 0.44 |
| COMPARE-ABSORB |  | BVS | 0.1 | 45.8 | 31.5 | 22.6 | 20.4 | 52 |  |  | 14.6 |  | 2.51 ± 0.50 |
|  |  | DES | 0.1 | 41.4 | 32.9 | 25.5 | 22.2 | 52.7 |  |  | 13.1 |  | 2.49 ± 0.49 |
| Andrew Kei-Yan Ng | 2021 | BVS | 0 | 55.4 | 14.9 | 23.8 |  |  |  |  |  |  |  |
|  |  | DES | 3.2 | 49.1 | 25.8 | 18.2 |  |  |  |  |  |  |  |
| FUTURE-II | 2021 | BVS |  | 58.4 | 22.2 | 19.5 | 6.8 | 2.8 |  |  |  |  | 2.90 ± 0.47 |
|  |  | DES |  | 51.8 | 27.9 | 20.4 | 4.4 | 3.7 |  |  |  |  | 2.92 ± 0.46 |
| Quentin de Hemptinne | 2022 | BVS |  | 60 | 36.7 | 3.3 |  | 33.3 |  |  |  |  |  |
|  |  | BP-DES |  | 63.3 | 33.3 | 3.3 |  | 30 |  |  |  |  |  |
|  |  | DP-DES |  | 63.3 | 36.7 | 0 |  | 30 |  |  |  |  |  |
| PRAGUE-22 | 2022 | BVS |  | 44 | 36 | 20 |  | 40 |  |  |  |  |  |
|  |  | DES |  | 52 | 28 | 20 |  | 44 |  |  |  |  |  |
| Piotr Rola | 2022 | BVS |  | 41.4 | 31.6 | 25.3 |  |  |  |  |  |  |  |
|  |  | BP-DES |  | 38.4 | 33.1 | 27.8 |  |  |  |  |  |  |  |
| Eriksen, Erlend | 2023 | BVS |  | 38.7 | 41.9 | 19.4 |  |  |  |  |  |  | 3.13 ± 0.46 |
|  |  | DES |  | 48.6 | 42.9 | 8.57 |  |  |  |  |  |  | 3.24 ± 0.43 |
| Joo Myung Lee | 2023 | BVS | 0.8 | 59.9 | 24.1 | 15.9 |  | 51.7 |  |  |  |  |  |
|  |  | DES | 18.2 | 74.4 | 24.1 | 8.5 |  | 66 |  |  |  |  |  |
| RIBS VI | 2024 | BVS |  | 46 | 34 | 19 |  |  |  |  |  |  | 2.69 ± 0.4 |
|  |  | DCB |  | 45 | 32 | 19 |  |  |  |  |  |  | 2.61 ± 0.5 |
|  |  | DES |  | 43 | 31 | 23 |  |  |  |  |  |  | 2.66 ± 0.5 |
| ISAR-RESORB | 2024 | BVS |  | 42.4 | 39 | 18.6 |  | 68.4 |  |  |  |  | 2.91 ± 0.43 |
|  |  | BP-DES |  | 46 | 28.6 | 25.4 |  | 75 |  |  |  |  | 2.92 ± 0.41 |
| Adrian Włodarczak | 2024 | BVS | 1.6 | 41.4 | 31.6 | 25.3 |  |  |  |  |  |  |  |
|  |  | BP-DES | 0.6 | 38.4 | 33.1 | 27.8 |  |  |  |  |  |  |  |
| Jun Li | 2025 | BVS |  | 49 | 19 | 22 |  |  |  |  |  |  |  |
|  |  | BP-DES |  | 43 | 28 | 29 |  |  |  |  |  |  |  |
| BIOHEART-II | 2025 | BVS |  | 43.7 | 40.5 | 15.3 | 0.9 |  |  |  |  |  | 2.91 ± 0.47 |
|  |  | DES |  | 47 | 30.9 | 21.7 | 3.2 |  |  |  |  |  | 2.97 ± 0.49 |
| IRONMAN II | 2025 | BVS |  | 57.8 | 14.2 | 28 | 14.6 | 3.5 |  |  |  |  |  |
|  |  | DES |  | 61.6 | 12.3 | 26.1 | 19.4 | 3.5 |  |  |  |  |  |

Supplementary Table 5. Procedural characteristics of included studies.

| **First Author/Study** | **Year** | **Device** | **Pre-dilation** | | | **Dilation** | | | | **Post-dilation** | | |
| --- | --- | --- | --- | --- | --- | --- | --- | --- | --- | --- | --- | --- |
|  |  |  | **Predilation**  **(%)** | **Balloon diameter**  **(mm)** | **Maximum pressure**  **(atm)** | **Number of stents/scaffolds**  **(n)** | **Stent/scaffold size**  **(mm)** | **Stent/scaffold length**  **(mm)** | **Pressure**  **(atm)** | **Post-dilation**  **(%)** | **Balloon diameter,**  **(mm)** | **Max inflation pressure(atm)** |
| Tommaso Gori | 2013 | BVS | 100 | 2.7±0.4 |  | 1.24 | 3.1±0.4 | 19.4±4.6 | 13.8±2.2 | 14 |  | 15.3±4.0 |
|  |  | DES | 100 | 2.54±0.3 |  | 1.26 | 3.1±0.4 | 17.9±6.9 | 13±2.4 | 14 |  | 12.2±3.9 |
| BVS-EXAMINATION | 2015 | BVS | 81 |  |  | 1.14 ± 0.39 | 3.22 ± 0.33 | 22.53 ± 8.80 |  | 36.3 |  |  |
|  |  | DES | 29 |  |  | 1.13 ± 0.37 | 3.19 ± 0.40 | 21.78 ± 9.17 |  | 15.2 |  |  |
| Bernardo Cortese | 2015 | BVS | 94.1 | 2.75 (2.5-3.0) |  |  | 3.0 (3.0-3.5) | 18 (18-28) | 10 (9-14) | 94.1 | 3.5 (3.0-4.0) |  |
|  |  | DES | 60 | 2.5 (2.0-2.5) |  |  | 3.0 (2.75-3.5) | 23 (18-33) | 14 (12-16) | 37.9 | 3.5 (3.0-3.75) |  |
| Charis Costopoulos | 2015 | BVS | 97.8 |  |  |  | 3.1±0.4 | 36.5±19.4 |  | 99.3 | 3.2±0.4 | 20.9±4.9 |
|  |  | DES | 75.8 |  |  |  | 3.1±0.4 | 19.4±23.1 |  | 77.4 | 3.1±0.4 | 19±6.0 |
| ABSORB III | 2015 | BVS |  |  |  |  | 3.18±0.43 | 20.5±7.2 | 15.4±3.0 | 65.5 |  |  |
|  |  | DES |  |  |  |  | 3.12±0.45 | 20.7±9.0 | 15.4±3.2 0.83 | 51.2 |  |  |
| ABSORB China | 2015 | BVS |  | 2.8 ± 0.4 |  |  | 3.1 ± 0.4 | 22.8 ± 6.7 | 12.4 ± 3.3 |  | 3.3 ± 0.4 | 16.8 ± 3.8 |
|  |  | DES |  | 2.7 ± 0.4 |  |  | 3.1 ± 0.4 | 22.3 ± 5.8 | 11.8 ± 3.3 |  | 3.2 ± 0.4 | 16.9 ± 3.4 |
| Hiroyoshi Kawamoto | 2015 | BVS |  | 99 |  | 2.1 ± 1.1 | 2.99 ± 0.31 | 50.8 ± 29.5 |  | 99.5 |  |  |
|  |  | DES |  | 79.5 |  | 2.1 ± 1.4 | 2.94 ± 0.41 | 54.6 ± 39.0 |  | 98 |  |  |
| Hiroyoshi Kawamoto | 2015 | BVS | 100 | 2.80+0.37 |  |  | 3.09+0.37 |  | 11.6+3.8 | 82.2 | 3.18+0.44 |  |
|  |  | DES | 100 | 2.86+0.36 |  |  | 3.13+0.38 |  | 11.9+3.7 | 77.4 | 3.29+0.51 |  |
| ABSORB II | 2015 | BVS | 100 |  |  |  | 3.01±0.31 |  |  | 61 | 3.08±0.34 | 14.23±3.43 |
|  |  | DES | 99 |  |  |  | 3.05±0.28 |  |  | 59 | 3.16±0.36 | 15.03±3.33 |
| BONITO | 2016 | BVS |  |  |  | 2.2±1.1 | 3.2±0.4 | 51.3±24.1 |  | 90.8 | 3.3±0.4 |  |
|  |  | DES |  |  |  | 2.1±1.0 | 3.1±0.5 | 54.2±28.6 |  | 78.6 | 3.2±0.5 |  |
| Daniel Dalos | 2016 | BVS | 85 | 2.64 ± 0.51 |  |  | 3.23 ± 0.34 | 21.80 ± 5.62 | 13 ± 2 | 55 | 3.41 ± 0.47 | 15 ± 3 |
|  |  | DES | 58 | 2.34 ± 0.46 |  |  | 3.07 ± 0.54 | 21.13 ± 9.21 | 12 ± 3 | 68 | 3.27 ± 0.60 | 16 ± 4 |
| José de Ribamar Costa Jr1 | 2016 | BVS | 99.7 |  |  |  | 2.97±0.6 | 23.4±9.8 |  | 68.8 |  |  |
|  |  | DES | 69.4 |  |  |  | 2.99± 0.1 | 23.8±12.1 |  | 42.2 |  |  |
| Jiang Ming Fam | 2016 | BVS | 54.1 | 2.54±0.47 |  | 1.28±0.61 | 3.21±0.33 | 26.32±13.27 |  | 39.7 | 3.45±0.41 |  |
|  |  | DES | 28.4 | 2.40±0.48 |  | 1.39±0.73 | 3.20±0.46 | 27.76±14.81 |  | 21.8 | 3.54±0.59 |  |
| Yoichi Imori | 2016 | BVS |  |  |  |  | 3.1 ± 0.4 | 20.8 ± 5.2 |  |  |  |  |
|  |  | DES |  |  |  |  | 3.0 ± 0.4 | 19.7 ± 5.1 |  |  |  |  |
| UNDERDOGS | 2016 | BVS | 98 | 2.93 ± 0.4 |  |  | 3 ± 0.4 | 54 ± 15 |  | 95 | 3.5 ± 0.5 |  |
|  |  | DES | 98 | 2.49 ± 0.2 |  |  | 3 ± 0.4 | 53 ± 14 |  | 66 | 3.45 ± 0.5 |  |
| V. F. Panoulas | 2016 | BVS | 100 |  |  |  | 2.85±0.35 | 46.1±19.7 |  | 100 | 3.3±0.4 | 21.1±4.5 |
|  |  | DES | 65 |  |  |  | 2.83±0.41 | 40.6±24 |  | 62.5 | 3.2±0.6 | 17.6 ±5. |
| TROFI II | 2016 | BVS | 55.8 |  |  | 1.2 ± 0.4 | 3.25 ± 0.30 | 20.6 ± 5.8 | 14.1 ± 3.8 | 50.5 | 3.51 ± 0.34 | 15.8 ± 3.4 |
|  |  | DES | 51 |  |  | 1.1 ± 0.4 | 3.12 ± 0.37 | 20.7 ± 6.7 | 13.3 ± 3.0 | 25.5 | 3.29 ± 0.62 | 18.6 ± 3.9 |
| Corrado Tamburino | 2016 | BVS |  |  |  |  |  |  |  | 51.9 |  |  |
|  |  | DES |  |  |  |  |  |  |  | 50.8 |  |  |
| Toru Naganuma | 2017 | BVS |  |  |  |  |  |  |  |  |  |  |
|  |  | DES |  |  |  |  |  |  |  |  |  |  |
| AIDA | 2017 | BVS | 98.6 |  |  | 1.54±0.84 | 2.73±0.27 | 31.1±19.6 |  | 74 |  |  |
|  |  | DES | 96.9 |  |  | 1.45±0.79 | 2.88±0.35 | 29.7±19.2 |  | 49.1 |  |  |
| Carlos M. Campos | 2017 | BVS |  |  |  |  |  |  |  | 74.8 |  |  |
|  |  | DES |  |  |  |  |  |  |  | 41.9 |  |  |
| Yaling Han | 2018 | BVS | 99.6 | 2.78 ± 0.44 | 11.7 ± 3.1 |  | 3.18 ± 0.37 | 20.2 ± 4.0 | 12.2 ± 2.6 | 83.5 | 3.26 ± 0.40 | 17.9 ± 3.3 |
|  |  | DES | 100 | 2.75 ± 0.43 | 11.4 ± 3.0 |  | 3.14 ± 0.37 | 19.6 ± 3.1 | 12.4 ± 2.8 | 74.2 | 3.26 ± 0.40 | 18.3 ± 3.6 |
| Jose M. de la Torre Hernandez | 2016 | BVS | 97.6 |  |  |  | 3.08±0.4 | 19.3±6 | 16±3 | 64.8 |  |  |
|  |  | DES | 25.4 |  |  |  | 3.01±0.5 | 20.6±8 | 15.4±3 | 38.4 |  |  |
| Andrea Cuculo | 2016 | BVS |  |  |  |  | 2.9±0.37 | 40.96±14.61 |  |  |  |  |
|  |  | DES |  |  |  |  | 2.83±0.43 | 44.65±15.73 |  |  |  |  |
| EVERBIO II | 2016 | BVS |  |  |  |  |  |  |  |  |  |  |
|  |  | DP-DES |  |  |  |  |  |  |  |  |  |  |
|  |  | BP-DES |  |  |  |  |  |  |  |  |  |  |
| Kugler, Christine | 2017 | BVS |  |  |  | 3.2 ± 1.3 | 3.3 ± 0.4 | 81.7 ± 29.1 | 18.6 ± 3.2 |  |  |  |
|  |  | DES |  |  |  | 3.0 ± 1.2 | 3.3 ± 0.5 | 79.3± 27.4 | 17.9 ± 2.3 |  |  |  |
| Frédéric Maes | 2018 | BVS | 83 |  |  | 2.6 ± 1.5 | 2.85 ± 0.35 | 21.3 ± 6.1 | 16 ± 3 | 71 |  |  |
|  |  | DES | 52 |  |  | 2.4 ± 1.3 | 2.95 ± 0.46 | 20.8 ± 8.3 | 16 ± 3 | 33 |  |  |
| BIORESOLVE-ISR | 2018 | BVS | 100 |  |  | 1.2 ± 0.4 |  | 28 (18–36) | 14.4 ± 4.4 | 86 |  |  |
|  |  | DES | 86 |  |  | 1.2 ± 0.5 |  | 28 (21–36) | 16.3 ± 4.5 | 66 |  |  |
| ABSORB IV | 2018 | BVS | 100 |  |  |  | 3.22±0.44 | 20.5±8.3 | 16.3 ± 3.1 | 84 |  |  |
|  |  | DES | 100 |  |  |  | 3.16±0.44 | 20.1±7.9 | 15.9±3.1 | 55 |  |  |
| Giuseppe Tarantini | 2018 | BVS | 97.6 |  |  |  |  |  |  | 86.4 |  |  |
|  |  | DES | 62.9 |  |  |  |  |  |  | 30.3 |  |  |
| ISAR-Absorb MI | 2018 | BVS | 95.3 |  |  | 1.0 ± 0.1 | 3.2 ± 0.4 | 25.7 ± 12.3 | 17.0 ± 4.0 | 56.6 |  |  |
|  |  | DES | 81.8 |  |  | 1.0 ± 0.0 | 3.2 ± 0.4 | 28.6 ± 15.5 | 16.0 ± 4.0 | 34.8 |  |  |
| COVER-AMI | 2019 | BVS | 90 |  |  |  | 3.3 ± 0.4 | 23.7 ± 5.2 |  | 80 |  |  |
|  |  | DES | 40 |  |  |  | 3.7 ± 0.4 | 21.6 ± 5.4 |  | 10 |  |  |
| Yizhe Wu | 2019 | BVS | 99 | 2.88 ± 0.46 | 12.06 ± 3.12 | 1.06 ± 0.29 | 3.15 ± 0.32 | 20.58 ± 5.11 | 12.48 ± 2.81 | 94.8 | 3.26 ± 0.38 | 16.89 ± 3.42 |
|  |  | DES | 91.2 | 2.77 ± 0.45 | 11.86 ± 3.31 | 1.02 ± 0.15 | 3.11 ± 0.31 | 21.79 ± 5.24 | 13.08 ± 2.90 | 73.1 | 3.22 ± 0.40 | 17.47 ± 3.45 |
| Piotr Desperak | 2020 | BVS | 95.4 |  |  | 1.32 ±0.59 | 3.06 ±0.38 | 21.69 ±5.67 | 13.16 ±2.52 | 60.9 |  |  |
|  |  | DES | 77.3 |  |  | 1.44 ±0.80 | 2.98 ±0.51 | 22.10 ±8.03 | 14.26 ±2.83 | 27.8 |  |  |
| Alexandre Hideo-Kajita | 2020 | BVS |  |  |  | 1.3 ± 0.7 | 3.3 ± 0.3 | 22.0 ± 4.5 |  |  |  |  |
|  |  | DES |  |  |  | 1.3 ± 0.6 | 3.2 ± 0.4 | 22.3 ± 6.4 |  |  |  |  |
| Yongcheol Kim | 2020 | BVS |  |  |  |  |  |  |  |  |  |  |
|  |  | DES |  |  |  |  |  |  |  |  |  |  |
| Jongkwon Seo | 2020 | BVS |  |  |  | 1.4 ± 0.6 |  | 32.0 ± 12.2 |  | 67 | 3.41 ± 0.35 | 18.1 ± 4.3 |
|  |  | DES |  |  |  | 1.3 ± 0.6 |  | 36.6 ± 14.2 |  | 54 | 3.18 ± 0.48 | 17.0 ± 4.0 |
| COMPARE-ABSORB |  | BVS | 96.5 | 3.0 (2.5-3.0) | 16 (12-18) | 1 (1-2) | 3.0 (2.8-3.5) | 28 (18-36) |  | 92.8 | 3.5 (3.0-3.5) | 18 (16-20) |
|  |  | DES | 78.6 | 3.0 (2.5-3.0) | 14 (12-16) | 1 (1-1) | 3.0 (2.8-3.5) | 28 (18-38) |  | 58 | 3.5 (3.0-3.5) | 18 (16-20) |
| Andrew Kei-Yan Ng | 2021 | BVS |  |  |  |  | 3.06 ± 0.36 | 18 (18–28) |  |  |  |  |
|  |  | DES |  |  |  |  | 2.90 ± 0.41 | 28 (18–44) |  |  |  |  |
| FUTURE-II | 2021 | BVS | 100 | 2.77 ± 0.44 | 12.5 ± 3.1 |  | 3.19 ± 0.46 | 21.1 ± 5.0 | 11.9 ± 2.4 | 95.5 | 3.35 ± 0.49 | 17.1 ± 2.9 |
|  |  | DES | 99.1 | 2.72 ± 0.43 | 12.1 ± 3.0 |  | 3.20 ± 0.46 | 21.6 ± 4.8 | 11.8 ± 2.6 | 81 | 3.40 ± 0.51 | 16.7 ± 2.9 |
| Quentin de Hemptinne | 2022 | BVS | 100 |  |  |  | 3.20 ± 0.25 | 22.0 ± 3.4 |  | 100 | 18.5 ± 2.4 |  |
|  |  | BP-DES | 73.3 |  |  |  | 3.22 ± 0.52 | 22.9 ± 6.2 |  | 70 | - |  |
|  |  | DP-DES | 83.3 |  |  |  | 3.24 ± 0.45 | 27.6 ± 10.0 |  | 63.3 | - |  |
| PRAGUE-22 | 2022 | BVS | 100 |  |  |  |  | 24.6 ± 10.7 |  | 92 |  |  |
|  |  | DES | 80 |  |  |  |  | 27.6 ± 11.1 |  | 72 |  |  |
| Piotr Rola | 2022 | BVS |  | 3.2 ± 0.3 | 17.7 ± 0.8 | 1.1 ± 0.2 | 3.28 ± 0.27 | 20.8 ± 3.3 |  |  | 3.5 ± 0.3 | 17.7 ± 0.8 |
|  |  | BP-DES |  | 3.1 ± 0.3 | 15.9 ± 1.9 | 1.2 ± 0.4 | 3.24 ± 0.31 | 23.9 ± 4.1 |  |  | 3.3 ± 0.3 | 16.7 ± 1.0 |
| Eriksen, Erlend | 2023 | BVS | 54.8 | 2.71 ± 0.30 | 11.1 ± 3.14 | 1.23 ± 0.43 | 3.13 ± 0.32 | 21.7 ± 1.49 | 15.1 ± 1.41 | 96.8 | 3.60 ± 0.38 | 17.3 ± 3.46 |
|  |  | DES | 48.6 | 2.64 ± 0.32 |  | 1.10.1 ± 2.8731 ± 0.58 | 3.26 ± 0.44 | 18.8 ± 1.40 | 15.4 ± 2.29 | 91.4 | 3.64 ± 0.37 | 17.4 ± 3.90 |
| Joo Myung Lee | 2023 | BVS |  |  |  | 1.2 ± 0.4 | 3.24 ± 0.30 | 24.6 ± 11.3 |  |  |  |  |
|  |  | DES |  |  |  | 1.0 ± 0.0 | 3.17 ± 0.47 | 33.7 ± 15.3 |  |  |  |  |
| RIBS VI | 2024 | BVS |  |  |  |  |  |  | 20 ± 4 |  |  |  |
|  |  | DCB |  |  |  |  |  |  | 18 ± 4 |  |  |  |
|  |  | DES |  |  |  |  |  |  | 20 ± 4 |  |  |  |
| ISAR-RESORB | 2024 | BVS | 98.3 |  |  |  | 3.2 ± 0.3 | 26.7 ± 12.1 |  | 88.1 | 3.5 ± 0.3 | 18.2 ± 4.5 |
|  |  | BP-DES | 85.7 |  |  |  | 3.2 ± 0.4 | 26.8 ± 10.9 |  | 65.1 | 3.4 ± 0.7 | 16.5 ± 4.8 |
| Adrian Włodarczak | 2024 | BVS |  | 3.2 ±0.3 | 17.7 ±0.8 | 1.1 ±0.2 | 3.28 ±0.27 | 20.8 ±3.3 |  |  | 3.5 ±0.3 | 17.7 ±0.8 |
|  |  | BP-DES |  | 3.1 ±0.3 | 15.9 ±1.9 | 1.2 ±0.4 | 3.24 ±0.31 | 23.9 ±4.1 |  |  | 3.3 ±0.3 | 16.7 ±1.0 |
| Jun Li | 2025 | BVS | 100 | 3.3 ± 0.25 | 22.6 ± 3.3 | 1.72 ± 0.38 |  |  |  |  |  |  |
|  |  | BP-DES | 100 | 3.3 ± 0.21 | 21.7 ± 2.5 | 1.21 ± 0.41 |  |  |  |  |  |  |
| BIOHEART-II | 2025 | BVS | 100 | 2.69 ± 0.51 | 12.2±2.8 |  | 3.27 ± 0.43 | 20.9 ± 4.9 | 11.9 ± 2.7 | 99.5 | 3.45 ± 0.45 | 17.4 ± 3.4 |
|  |  | DES | 100 | 2.68 ± 0.48 | 12.1±2.7 |  | 3.29 ± 0.42 | 21.2 ± 5.0 | 11.8 ± 2.6 | 90.3 | 3.41 ± 0.46 | 17.4 ± 3.1 |
| IRONMAN II | 2025 | BVS | 100 |  |  |  | 3.26 ± 0.43 | 24.8 ± 7.0 | 11.9 ± 1.90 | 91.4 |  | 17.0 ± 3.5 |
|  |  | DES | 99.6 |  |  |  | 3.21 ± 0.42 | 24.7 ± 7.2 | 11.5 ± 2.23 | 91.8 |  | 17.4 ± 3.2 |

Supplementary Fig. 1. Risk of bias assessment of included randomized controlled trials using the Cochrane RoB 2 tool.

Supplementary Fig. 2. Summary of risk of bias across included randomized controlled trials using the Cochrane RoB 2 tool.

Supplementary Table 6. Newcastle–Ottawa Scale (NOS) quality assessment of included cohort studies

| **Study (Author)** | **Year** | **Selection** | | | | **Comparability** | **Outcome** | | | **Total NOS Score** |
| --- | --- | --- | --- | --- | --- | --- | --- | --- | --- | --- |
|  |  | **Representativeness of exposed cohort** | **Selection of non-exposed cohort** | **Ascertainment of exposure** | **Outcome not present at start** | **Comparability of cohorts** | **Outcome assessment** | **Adequate follow-up duration** | **Adequacy of follow-up** |  |
| Tommaso Gori | 2013 | ★ | ★ | ★ | ★ | ☆ | ★ | ★ | ★ | **7** |
| BVS-EXAMINATION | 2015 | ★ | ★ | ★ | ★ | ★★ | ★ | ★ | ★ | **9** |
| Bernardo Cortese | 2015 | ★ | ★ | ★ | ★ | ★★ | ★ | ★ | ★ | **9** |
| Charis Costopoulos | 2015 | ★ | ★ | ★ | ★ | ★★ | ★ | ★ | ★ | **9** |
| Hiroyoshi Kawamoto | 2015 | ★ | ★ | ★ | ★ | ★★ | ★ | ★ | ★ | **9** |
| BONITO | 2016 | ★ | ★ | ★ | ★ | ★★ | ★ | ★ | ★ | **9** |
| Daniel Dalos | 2016 | ★ | ★ | ★ | ★ | ☆ | ★ | ★ | ★ | **7** |
| José de Ribamar Costa Jr | 2016 | ★ | ★ | ★ | ★ | ★★ | ★ | ★ | ★ | **9** |
| Jiang Ming Fam | 2016 | ★ | ★ | ★ | ★ | ★★ | ★ | ★ | ★ | **9** |
| Yoichi Imori | 2016 | ★ | ★ | ★ | ★ | ★★ | ★ | ★ | ★ | **9** |
| UNDERDOGS | 2016 | ★ | ★ | ★ | ★ | ★★ | ★ | ★ | ★ | **9** |
| V. F. Panoulas | 2016 | ★ | ★ | ★ | ★ | ★★ | ★ | ★ | ★ | **9** |
| Corrado Tamburino | 2016 | ★ | ★ | ★ | ★ | ★★ | ★ | ★ | ★ | **9** |
| Andrea Cuculo | 2016 | ★ | ★ | ★ | ★ | ★★ | ★ | ★ | ★ | **9** |
| Toru Naganuma | 2017 | ★ | ★ | ★ | ★ | ★★ | ★ | ★ | ★ | **9** |
| Carlos M. Campos | 2017 | ★ | ★ | ★ | ★ | ★★ | ★ | ★ | ★ | **9** |
| Kugler, Christine | 2017 | ★ | ★ | ★ | ★ | ★★ | ★ | ★ | ★ | **9** |
| Frédéric Maes | 2018 | ★ | ★ | ★ | ★ | ★★ | ★ | ★ | ★ | **9** |
| BIORESOLVE-ISR | 2018 | ★ | ★ | ★ | ★ | ★★ | ★ | ★ | ★ | **9** |
| Giuseppe Tarantini | 2018 | ★ | ★ | ★ | ★ | ★★ | ★ | ★ | ★ | **9** |
| Piotr Desperak | 2020 | ★ | ★ | ★ | ★ | ☆ | ★ | ★ | ★ | **7** |
| Alexandre Hideo-Kajita | 2020 | ★ | ★ | ★ | ★ | ☆ | ★ | ★ | ★ | **7** |
| Yongcheol Kim | 2020 | ★ | ★ | ★ | ★ | ★★ | ★ | ★ | ★ | **9** |
| Andrew Kei-Yan Ng | 2021 | ★ | ★ | ★ | ★ | ★★ | ★ | ★ | ★ | **9** |
| Quentin de Hemptinne | 2022 | ★ | ★ | ★ | ★ | ★★ | ★ | ★ | ★ | **9** |
| PRAGUE-22 | 2022 | ★ | ★ | ★ | ★ | ☆ | ★ | ★ | ★ | **7** |
| Piotr Rola | 2022 | ★ | ★ | ★ | ★ | ☆ | ★ | ★ | ★ | **7** |
| Joo Myung Lee | 2023 | ★ | ★ | ★ | ★ | ★★ | ★ | ★ | ★ | **9** |
| RIBS VI | 2024 | ★ | ★ | ★ | ★ | ★★ | ★ | ★ | ★ | **9** |
| Adrian Włodarczak | 2024 | ★ | ★ | ★ | ★ | ☆ | ★ | ★ | ★ | **7** |
| Jun Li | 2025 | ★ | ★ | ★ | ★ | ★ | ★ | ★ | ★ | **8** |

Supplementary Fig. 3. Forest plots of additional clinical outcomes comparing bioresorbable scaffolds versus drug eluting stents.

Supplementary Fig. 4. Two-year major adverse cardiovascular events and key device-related outcomes comparing bioresorbable scaffolds versus drug eluting stents.

Supplementary Fig. 5. Two-year mortality and vessel-related outcomes comparing bioresorbable scaffolds versus drug eluting stents.

Supplementary Fig. 6. Three-year major clinical outcomes (BVS vs DES).

Supplementary Fig. 7. Three-year additional clinical outcomes (BVS vs DES).

Supplementary Fig. 8. Five-year MACE, thrombosis, MI, and TLR comparing bioresorbable scaffolds versus drug-eluting stents.

Supplementary Fig. 9. Five-year additional clinical outcomes (death, cardiac death, TVR, TVF, and target-vessel MI) comparing bioresorbable scaffolds versus drug-eluting stents.

Supplementary Fig. 10. Pooled angiographic outcomes comparing bioresorbable vascular scaffolds versus drug-eluting stents at follow-up.

Supplementary Fig. 11. Prespecified subgroup analyses of 1-year major adverse cardiac events (MACE) comparing bioresorbable scaffolds versus drug-eluting stents.

Supplementary Fig. 12. Sensitivity analysis of 1-year MACE according to endpoint definition (device-oriented composite endpoint [DOCE] vs patient-oriented composite endpoint [POCE]) comparing bioresorbable scaffolds versus drug-eluting stents.

Supplementary Fig. 13. Subgroup analysis of 1-year major adverse cardiovascular events (MACE) according to study design (randomized controlled trials vs cohort studies) comparing bioresorbable scaffolds versus drug-eluting stents.

Supplementary Fig. 14. Funnel plots for publication bias assessment of 1-year clinical outcomes comparing bioresorbable scaffolds versus drug-eluting stents.

Supplementary Fig. 15. Leave-one-out influence analyses of 1-year clinical outcomes comparing bioresorbable scaffolds versus drug-eluting stents.

Supplementary Fig. 16. Funnel plots assessing small-study effects for angiographic outcomes.

Supplementary Table 7. Egger’s regression test for publication bias across analyzable outcomes

| **Outcome** | **No. of studies** | **Egger’s test *P* value** | **Interpretation** |
| --- | --- | --- | --- |
| MACE | 38 | 0.852 | No evidence of small-study effects |
| Death | 27 | 0.802 | No evidence of small-study effects |
| Cardiac death | 33 | 0.367 | No evidence of small-study effects |
| MI | 28 | 0.957 | No evidence of small-study effects |
| TLR | 36 | 0.557 | No evidence of small-study effects |
| TVR | 24 | 0.221 | No evidence of small-study effects |
| TVF | 9 | 0.193 | Interpret cautiously, fewer than 10 studies |
| Thrombosis | 28 | 0.232 | No evidence of small-study effects |
| TV-MI | 19 | 0.542 | No evidence of small-study effects |
